# Differential neuropilin isoform expressions highlight plasticity in macrophages in the heterogenous TME through *in-silico* profiling

**DOI:** 10.3389/fimmu.2025.1547330

**Published:** 2025-03-11

**Authors:** Hyun-Jee Han, Marcos Rubio-Alarcon, Thomas Allen, Sunwoo Lee, Taufiq Rahman

**Affiliations:** ^1^ Department of Pharmacology, University of Cambridge, Cambridge, United Kingdom; ^2^ Institute for Medical Research, University of Cambridge, Cambridge, United Kingdom; ^3^ Department of Medical Genetics, University of Cambridge, Cambridge, United Kingdom

**Keywords:** neuropilin (NRP), macrophage, single cell profiling, TME (tumor microenvironment), ccRCC, SKCM

## Abstract

**Introduction:**

The nuanced roles of neuropilin (NRP) isoforms, NRP1 and NRP2, have attracted considerable scientific interest regarding cancer progression. Their differential expressions across various cancer types are specific to NRP isoforms which are shown in a cancer type-dependent manner. It accounts for the different mechanisms involved, driven by a co-expression of gene-sets associated with overexpressed *NRP1* or *NRP2*. Their different expressions on tumour-associated macrophages (TAMs) with disparate markers are associated with the heterogenous tumour microenvironment (TME) through their plasticity and pro-tumorigenic activities.

**Methods:**

Single-cell RNA sequencing (scRNA-seq) analyses were performed on tumours from clear cell Renal Cell Carcinoma (ccRCC) and skin cutaneous melanoma (SKCM) which exhibit the highest expressions of NRP1 and NRP2, respectively. Datasets were processed using established bioinformatics pipelines, including clustering algorithms, to determine cellular heterogeneity and quantify NRP isoform expression within distinct macrophage populations. Using differential gene expression analysis (DEGA) alongside co-enrichment studies, we explored gene-sets associated with NRP1 or NRP2 overexpression in TAMs.

**Results:**

Our analysis revealed a marked upregulation of *NRP1* in TAMs isolated from ccRCC and elevated *NRP2* expression in SKCM-derived TAMs. Both *NRP1^+^* and *NRP2^+^* macrophages showed an M2-like polarisation characterised by immune suppression and extracellular matrix degradation. Coupled with the previously uncharacterised *NRP isoform specific*- subpopulations within these cancers identified by DEGA, co-enrichment analyses demonstrated that the upregulation of gene-sets associated with *NRP1* is associated with angiogenesis and tumour progression through VEGF signalling, while gene-sets with *NRP2* showed dual functionality in the TME-dependent manner. Their distinct roles in regulating macrophage plasticity, tumour invasion, and metastasis were highlighted.

**Discussion:**

These findings underscore distinct isoform-specific mechanisms by which NRP1 and NRP2 contribute to TAM-mediated cancer progression. This study aims to establish a foundation for future research, leading to biological experiments with focused gene-sets derived from our findings. This approach can contribute to the development of immunomodulatory strategies targeting specific NRP isoforms in macrophages, tailored to individual cancer types and abnormal expressions of those gene markers, potentially offering a more effective therapeutic approach compared to broad-spectrum *NRP* inhibition strategies.

## Introduction

The tumour microenvironment (TME) is a complex ecosystem where immune cells, stromal cells, and extracellular components interact to shape cancer progression. Among those, macrophages have been causally associated with tumorigenesis and various stages of tumour progression and metastasis. High infiltration of tumour associated macrophages (TAMs) is a predictor of poor clinical outcome in various cancers (1, 2). TAMs play a pivotal role in this dynamic landscape, exhibiting remarkable plasticity that enables them to adopt either anti-tumorigenic (M1-like) or pro-tumorigenic (M2-like) phenotypes depending on microenvironment-derived factors and complex molecular profiles (3, 4). The heterogeneity of TAM populations across cancer types also adds another layer of complexity.

Recently, there has been a surge in interest surrounding the inhibition of neuropilins (NRPs) notably attributable to its aptness for impeding both SARS*-*CoV*-*2 cellular ingress (5) and neuropathic pain propagation resulting from NGF/TrkA signalling (6). Amongst the multifaceted effects associated with NRP, its implications in cancer progression driven by angiogenesis, cellular migration, invasion, and proliferation have prompted numerous investigations (7–12). NRP is a non*-*tyrosine kinase receptor, comprising two isoforms, neuropilin*-*1 (NRP1) and neuropilin*-*2 (NRP2) (13). While NRP1 and NRP2 share a 44% sequence identity across all domains, their structural resemblance is particularly striking in the large N*-*terminal extracellular region comprising 5 domains known as ‘a1’, ‘a2’, ‘b1’, ‘b2’, and ‘c’ (835 amino acid residues for NRP1 and 844 for NRP2), followed by a short transmembrane domain (23 residues for NRP1 and 25 for NRP2) and a compact cytoplasmic tail (44 residues for NRP1 and 42 for NRP2) (14–17). Notably, the b1b2 fragment within the N*-*terminal extracellular region exhibits a 50% sequence homology between NRP1 and NRP2, with remarkable overall 3D structural similarity (r.m.s.d ~ 2.3 Å over 307 Cα atoms) observed when they are overlaid onto each other (18). While the b1 domain appears to be structurally almost indistinguishable between the two NRP isoforms (r.m.s.d = 0.6 Å), the b2 domains superimpose less well due to the different conformations around the spikes (r.m.s.d. = 2.7 Å), contributing to interactions with different binding partners between NRP1 and NRP2 (18).

Beyond their established roles, NRPs are increasingly recognised for their influence on macrophage behaviour. Miyauchi et al. revealed that genetic ablation or pharmacologic manipulation of *NRP1* expression in microglia or bone marrow-derived macrophages arrested glioma progression and increased antitumorigenic polarization in these cells (19). Additionally, depletion of *NRP2* from TAMs impaired the clearance of apoptotic tumour cells and increased secondary necrosis within tumours (2). These findings suggest that NRP isoforms may be part of molecular switches that dictate TAM polarisation and function. Although NRPs have been implicated in cancer progression, the specific roles of each isoform in TAM polarisation through differentially regulated *NRP1* and *NRP2*-associated genes in cancers have not been explored at the single-cell level.

Our analysis of cell-specific expression patterns revealed that polarized macrophages consistently showed significantly increased levels of *NRP* compared to healthy samples, with different isoforms expressed depending on the cancer types, namely ccRCC and SKCM. Correlation analyses between *NRP*-related genes and differentially expressed genes (DEGs) directed subsequent enrichment analyses, providing insight into the distinct roles of *NRP* isoforms in macrophages across two diverse cancer contexts.

Although the expression pattern of *NRPs* in macrophages has been known, their differential expressions and functional implications in modulating the TME with cancer heterogeneity remain unclear. Here, we delve into the resulting phenotypic heterogeneity arising from distinct *NRP* gene expressions, elucidating the emergence of predicted functionally discrete TAM subpopulations within tumours, each characterised by related gene-sets. Hereby we confirm certain gene-sets in the *NRP* isoform-specific manner: *CTSL* and *CTSB* are associated with *NRP1* in TAM of ccRCC, as well as *PLTP, SDC3*, and *MMP14* which showed elevated expressions coupled with *NRP2* in the SKCM samples, adding to the array of effector mechanisms used by TAMs to aid tumour progression, as predicted based on the literature.

These findings provide evidence for a unifying framework where NRP isoforms are associated with TAM plasticity and contribute to cancer-specific heterogeneity in the TME. This will be important for the target validation with the identification of gene-sets in correlation with *NRP1* and *NRP2* which can work as biomarkers for cancer diagnosis and prognosis as therapeutic targets to reprogram TAMs and disrupt their pro-tumorigenic functions. Strategic designs of therapeutics should be tailored to precise oncogenic targets, as opposed to the pursuit of pan-NRP inhibitors lacking specificity which will indiscriminately impact multiple biological pathways.

## Results

### Differential expressions of NRP isoforms with tumour heterogeneity

**Figure 1:**
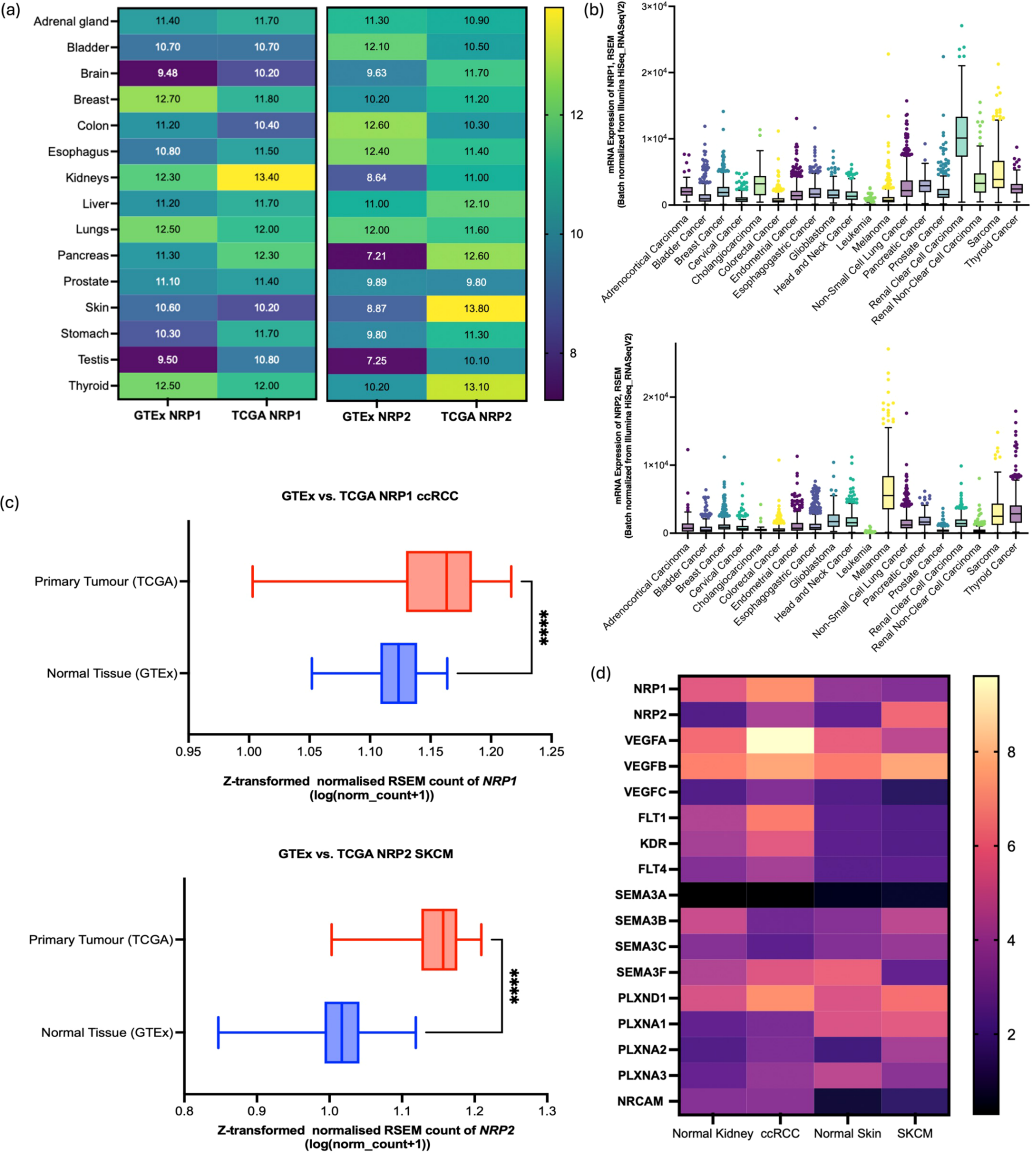
GTEx and TCGA analysis for the comparison between NRP1 and NRP2 expressions. **(a)** Comparative analysis of *NRP1* and *NRP2* expression levels across various tissues under healthy and cancerous conditions. Data sourced from GTEx and TCGA databases. **(b)** *NRP1* (upper panel) and *NRP2* (lower panel) mRNA expressions across the cancer tissues (RNA-seq RSEM, log2 (norm count + 1)) in TCGA datasets. Box and whisker plots with turkey whiskers representing medians and quartiles. **(c)** Differential expression of *NRP1in* ccRCC (upper panel) and *NRP2* in SKCM (lower panel) between the normal tissue and primary tumours within the TCGA TARGET GTEx cohort (n=912 and 1024, respectively). The box plot represents the Z-transformed normalised count of the gene expression in RSEM. The central line in each box denotes the median expression level, with the top and bottom edges of the box representing the interquartile range (IQR), **** represents p < 0.001. **(d)** Heatmap to compare the gene expression level in log2(TPM+1) highlighted in STRING in normal kidney, ccRCC, normal skin, and SKCM samples through matching between GTEx and TCGA datasets following normalisation. Yellow represents high expression; purple, low expression. Data collected from GEPIA2 web server (http://gepia2.cancer-pku.cn).

Analysis of TCGA samples revealed higher transcript levels of *NRP1* and *NRP2* compared to GTEx samples across a majority of the tissue types (**Figure 1a**). Furthermore, distinctive mRNA expression patterns for *NRP1* and *NRP2* were noted across the 19 distinct cancer types. Amongst the 19 cancer cell types, ccRCC cases showed the highest mRNA of *NRP1*, while *NRP2* was mostly expressed in SKCM samples (**Figure 1b**). One-way ANOVA with Dunnett’s multiple comparisons test revealed that the differences of mean RSEM of *NRP1* between ccRCC (RSEM = 10,391) and other cancer types were significant (*p* < 0.0001), and that of mean RSEM of *NRP2* expressed in SKCM (RSEM = 6,475) compared to other cancer types also showed a significant difference (*p* < 0.0001) (**Supplementary Table S1**). Through the comparison of mRNA levels in primary tumour samples to those in normal tissue from a combined cohort consisting of TCGA and TARGET GTEx samples, a distinct expression pattern emerged for *NRP1* where both the normal tissue and primary tumour samples showed a similar median expression level around the baseline, with primary tumours exhibiting a slightly higher median expression value. For *NRP2*, a marked increase in mRNA levels was observed in primary SKCM tumour samples compared to normal tissue (**Figure 1c**).

Specific genes associated with each isoform identified by STRING were used to examine their expression levels in ccRCC samples and SKCM samples through bulk-seq analysis using the TCGA dataset. *NRP1* - associated genes such as *VEGFA, FLT1, KDR, SEMA3F, PLXND1* exhibited higher mRNA expression levels in ccRCC samples compared to SKCM samples (**Figure 1d**). Similarly, *NRP2*-related genes (*SEMA3A, SEMA3B, SEMA3C, PLXNA1*) were expressed at higher levels in SKCM samples than in ccRCC samples. These differences may be attributable to specific expression patterns among heterogeneous cell types and their mechanistic pathways involving other gene sets.

### Differential expressions of the *NRP* isoforms in the occurrence of cancer compared to healthy samples at the single cell level

By using sc-RNAseq, this paper highlights the inter- and intra-tumoural heterogeneity of *NRP1* and *NRP2* expressions across different cell types in different cancer types, namely ccRCC and SKCM where each of the isoforms were distinctively highest.

**Figure 2:**
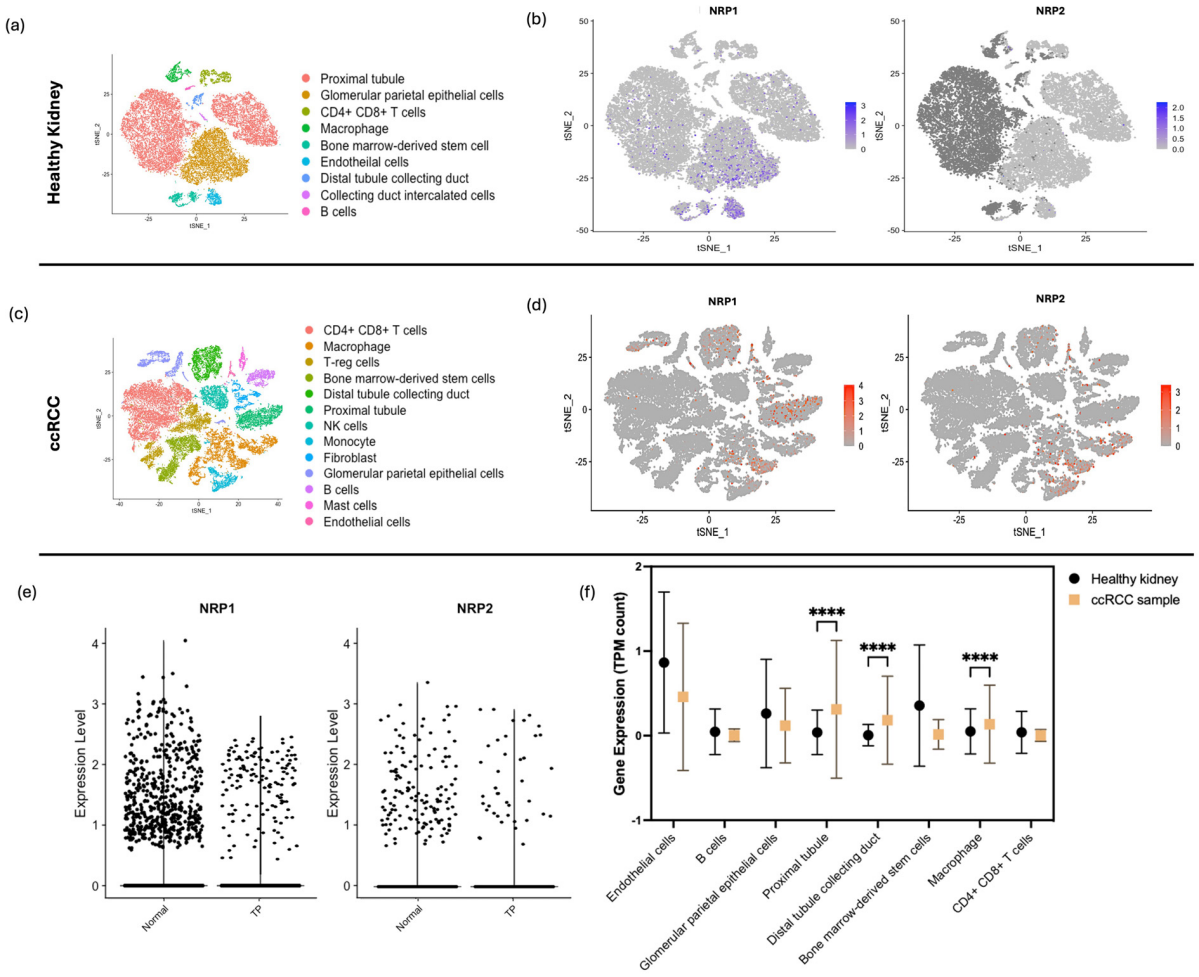
sc-RNAseq Analysis of *NRP1* and *NRP2* in Healthy and ccRCC Kidney Tissues. **(a)** tSNE plot displaying 9 distinct cell populations identified within kidney tissues from three healthy individuals, with each cluster differentiated by colour and annotated with specific cell type labels. **(b)** Feature plot representation on tSNE plots illustrating the expression of *NRP1* and *NRP2*; cell expression is indicated by blue intensity, representing a spectrum of expression levels. cells consisting of *NRP1* and *NRP2* markers were highlighted in a range of expression levels in blue from the normalised level 0 to 3 for *NRP1* and from 0 to 2 for *NRP2.* **(c)** tSNE plot showing 13 cell clusters derived from ccRCC patient samples, categorised by cell types, highlighting the diversity of kidney-specific and immune cells across all collected lesions. **(d)** Feature plots on tSNE maps for *NRP1* and *NRP2* expression in ccRCC samples, with varying intensities of red denoting expression levels. **(e)** Comparative violin plots of NRP1 and NRP2 expression levels across the tumour programmed (TP) and normal clusters, elucidating the association between *NRP* isoform expression and tumour-specific gene programmes. **(f)** Levels of *NRP1* expression compared between the healthy kidney sample and ccRCC sample across the different cell types through the TPM counts calculated through sc-RNAseq run on R. Two-way ANOVA performed with Bonferroni multiple comparison test. *P-*value indicated with **** <0.0001.

After quality control for the healthy kidney sample scRNA-seq dataset, a total of 23,366 high*-*quality cells were retained altogether for the downstream analysis (**Figure S2**). Clustering with a resolution parameter of 0.3 resulted in 9 distinct cell clusters which were visualised using tSNE plots based on principal component analysis (PCA) (**Figure 2a**). Cell clusters were classified according to the marker genes (**Supplementary Table S2**). In the feature plots, cells expressing high levels of *NRP1* and *NRP2* were predominantly observed in the regions indicating glomerular parietal epithelial cells, endothelial cells, proximal straight tubule cells, and macrophages (**Figure 2b**).

Based on rigorous quality control measures, 26771 cells with 25728 features were obtained from the selected ccRCC sample (**Supplementary Figure S2**). The tSNE analysis revealed 13 distinct cellular clusters (**Figure 2c**). As anticipated, the cellular composition of tumor tissues presented noticeable variability across the patients as compared to their matched pair of healthy-adjacent samples. Compared to the healthy kidney sample, ccRCC sample showed a higher level of *NRP1* and *NRP2* across a higher number of cell types, as distributed across the tSNE maps (**Figure 2d**). ccRCC samples exhibited elevated *NRP1* expression levels in specific cell types compared to those from healthy kidney tissues, as quantified by transcript count.

Based on the identified malignant gene expressions according to Bi et al. (20), sub*-*clusters with cancer cell*-*specific genes (**Supplementary Figure S3**) were identified and were visualised through the tSNE plots as ‘tumour programmed (TP)’ and ‘normal’ clusters (**Figure 2e**). Clusters consisting of the identified gene markers for TP revealed higher expressions of both *NRP1* and *NRP2* than the ‘normal’ cluster, yet *NRP1* at a distinctively higher level in the TP than *NRP2* (**Figure 2f**). A consistent feature across all tumor samples was the pronounced infiltration of immune cells within the TME, accompanied by its enrichment in the specialised kidney-specific epithelial and endothelial cell populations such as the proximal tubule and intercalated collecting duct tissues, which was the feature highlighted by TP (**Figure 2g**). Unexpectedly, a marked increase in *NRP1* expression was also observed in macrophages (*P* < 0.001) (**Supplementary Table S4**).

**Figure 3:**
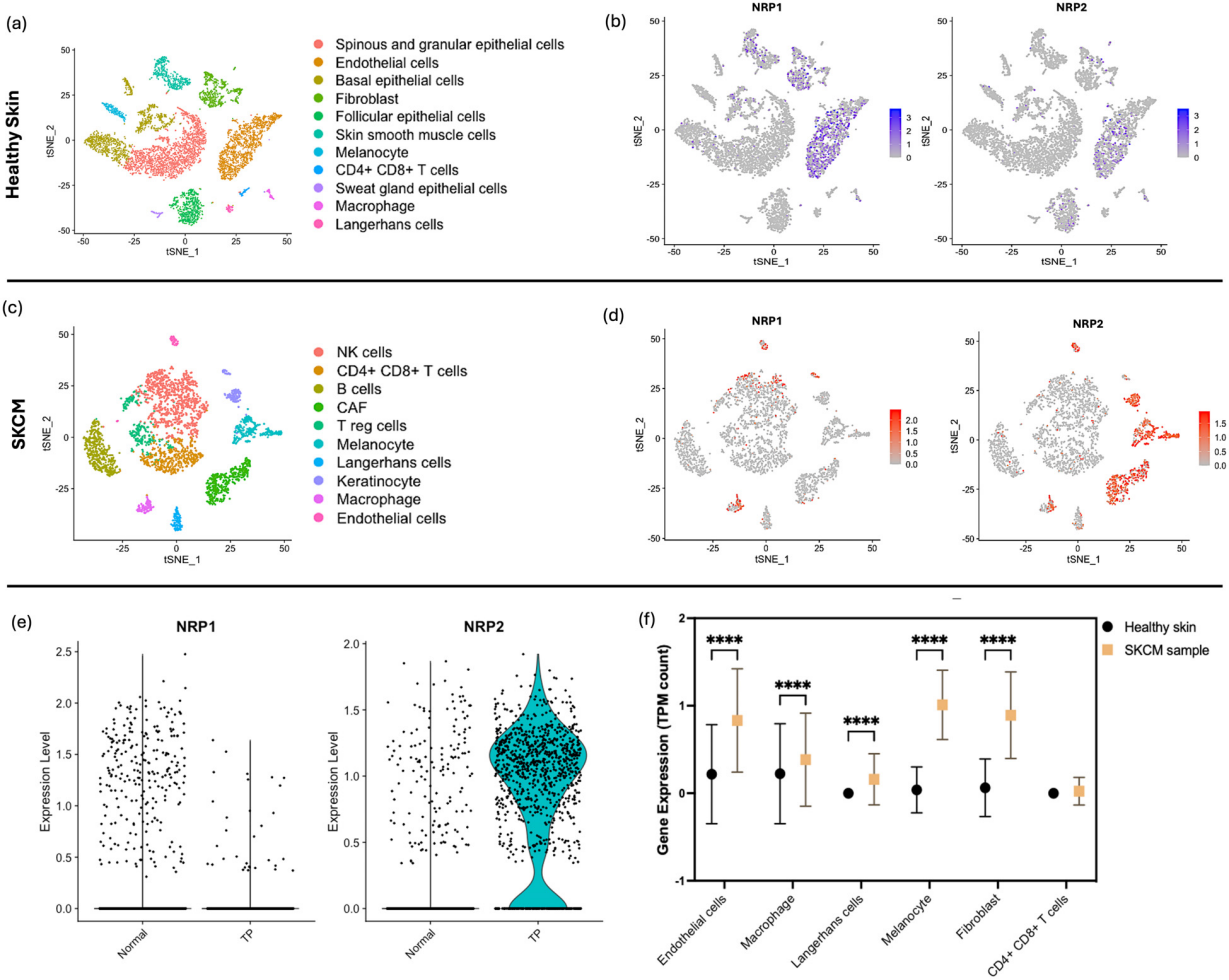
sc-RNAseq Analysis of NRP1 and NRP2 in Healthy and SKCM skin Tissues. **(a)** tSNE plot displaying 11 distinct cell populations from 84,363 cells identified within skin tissues from healthy individuals, with each cluster differentiated by colour and annotated with specific cell type labels. **(b)** Feature plot representation on tSNE plots illustrating the expression of *NRP1* and *NRP2*; In the feature plots, cells expressing *NRP1* and *NRP2* in each cell cluster were delineated by blue dots, with normalised expression intensity levels ranging from 0 to 3. **(c)** tSNE plot showing 10 cell clusters derived from SKCM patient samples, categorised by cell type, highlighting the diversity of skin-specific and immune cells across all collected lesions. **(d)** Feature plots on tSNE maps for *NRP1* and *NRP2* expression in SKCM samples, with varying intensities of red denoting expression levels. **(e)** Comparative violin plots of *NRP1* and *NRP2* expression levels in the normal and TP clusters, elucidating the association between *NRP* isoform expression and tumour^-^specific gene programmes. **(f)** Levels of *NRP2* expression compared between the healthy skin sample and SKCM sample across the different cell types through the TPM counts calculated through sc-RNAseq run on R. Two-way ANOVA performed with Bonferroni multiple comparison test. *P-*value indicated with **** <0.0001.

Sc-RNAseq data of 84,363 cells derived from healthy skin tissues was downloaded (GSM4850587) (21) which was processed for QC prior to analysis (**Supplementary Figure S3**). Clustering of the datasets led to 11 groups (**Figure 3a**). In addition, 22,846 genes from the SKCM data of 4,646 samples derived from SKCM patients skin tissues was downloaded (22) which was processed for QC prior to analysis (**Supplementary Figure S3**). A multitude of heterogeneous cell populations were unequivocally distinguished through PCA and tSNE plotted 10 disparate cell clusters which were identified with a resolution of 0.5 by examining expression patterns of canonical marker genes (**Supplementary Table S3**).

In alignment with observations from the healthy kidney sample, the healthy skin sample demonstrated elevated expression of *NRP1*, with a particular enrichment in endothelial cells and fibroblasts relative to *NRP2* (**Figure 3b**). Contrastingly, an increased level of *NRP2* became notable in the SKCM sample, including immune cells which are mostly found in the dermis, stromal cells, and skin*-*specific epithelial cells in the epidermis of skin (**Figure 3c**). Canonical cancer gene markers previously identified by Guan (23) were utilised to discern their distributions within the cell populations (**Supplementary Figure S5**). Clusters of cells which express a high level of those markers were highlighted in green in the tSNE map to pinpoint the specific cell clusters affected by SKCM (**Figure 3d**). A subsequent investigation into the comparison between the *NRP* isoforms for the expression levels between the SKCM cells of the TP and normal clusters highlights that *NRP2* is notably increased in the presence of tumour compared to *NRP1* (**Figure 3e**). However, the phenomenon was the opposite with *NRP1* where the gene was expressed more in the normal sample than the TP sample (**Figure 3f**), possibly due to the functional implication of *NRP1* in the skin dermal tissue for angiogenesis. Notably, we observed a significant increase in *NRP2* expression within macrophages, mirroring findings from healthy kidney samples and ccRCC, along with skin-specific cells such as Langerhans cells and melanocytes (**Supplementary Table S5**).

The diverse TME landscapes observed across patients in our study, as well as in other ccRCC and SKCM studies, suggest that patient stratification may be more effectively based on the abundance of specific cellular phenotypes within the TME rather than on patient-specific phenotypes. This highlights the need to reassess biomarker selection strategies to enhance personalized treatment approaches for ccRCC and SKCM.

Macrophage was the one which showed a significant increase in the NRP expression in the occurrence of cancer, and also amongst the cell types in tumour. Analysis of scRNA-seq data revealed a striking enrichment of NRP1 in macrophages from ccRCC samples, while NRP2 was predominantly expressed in macrophages from SKCM. These patterns were validated across multiple datasets, with significantly elevated transcript levels of NRP1 and NRP2 in tumour samples compared to healthy tissues (p<0.001).

### Macrophage plasticity and polarisation in ccRCC and SKCM correlated with higher *NRP* expressions

Following from the previous cell clustering, gene-sets from the cells clustered under ‘macrophage’ were extracted from each normal tissues and cancer types to understand the upregulation of specific genes.

**Figure 4:**
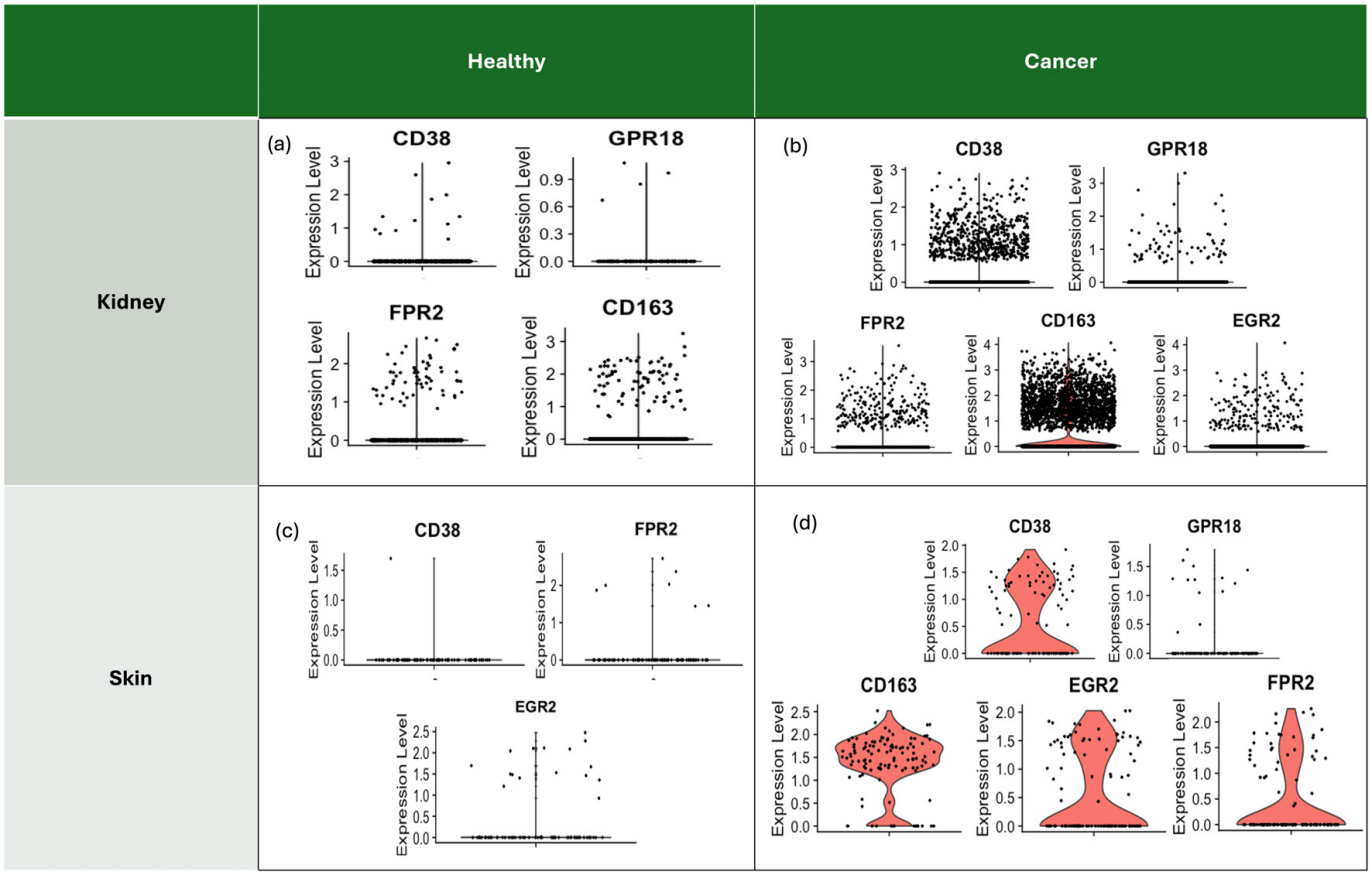
Signature markers for macrophages with polarisation to indicate the state of macrophages in each sample. Markers such as CD38, GPR18, FPR2 are known for the result of polarisation, and Type 2 macrophages (M2) after polarisation express upregulated gene markers such as CD163 and EGR2, which are shown as a consequence of polarisation in the TME, associated with TAM. Some samples did not even have a moderate level of mRNA to be quantified and plotted for the expression level. **(a)** Expression levels of gene markers of macrophages for the healthy kidney reveal low levels of the gene markers, implying a low possibility of the macrophage state being M2 TAM. **(b)** Overall upregulated expression levels of the gene markers of macrophages, particularly CD163, in the ccRCC sample indicate the likelihood of the macrophage state being polarised M2 TAM. **(c)** Expression levels of gene markers of macrophages for the healthy skin sample reveal low levels of the gene markers, even with an absence of CD163 expression, which is the main marker for TAM. **(d)** Overall upregulated expression levels of the gene markers of macrophages, particularly CD163 and EGR2, in the SKCM sample indicate the likelihood of the macrophage state being polarised M2 TAM.

Polarised tumour-associated macrophages show M1 and M2 types with heterogenous markers. Through the expression levels of signature markers indicative of macrophage polarisation states in various samples, TAMs of those samples were classified. Signatures such as CD38, GPR18, and FPR2 are associated with macrophage activation, while CD163 and EGR2 are known to be upregulated following M2 TAM polarisation. In both healthy kidney (**Figure 4a**) and skin (**Figure 4c**) tissues, the expression levels of M2 TAM markers were consistently low or absent. Notably, CD163, a primary marker for TAMs, was not detected in healthy skin, suggesting a lack of significant macrophage polarisation under normal conditions. On the other hand, in cancer samples, elevated expression levels of macrophage markers, particularly CD163, suggest a predominance of M2 TAMs within the tumour microenvironment (TME) of ccRCC (**Figure 4b**). Similarly, significant upregulation of CD163 and EGR2 highlights the polarised state of macrophages towards an M2 phenotype in melanoma samples (**Figure 4d**).

M2 TAM, known to show the highest expression of complement system C1Q genes, was also highlighted through their enrichment (**Supplementary Figure S7**). C1Q genes are products known to promote tumour progression in cancer by interacting with tumour-produced complement system molecules (24)In addition, tumour cells which were predicted to communicate with TAMs through the immune checkpoint *HLA-G – LILRB1/2* axis that shows involvement in polarisation into the immunosuppressive M2 phenotype and immune escape of the tumour (25) showed high correlations with *NRP1* and *NRP2* in the TCGA data of ccRCC (*P-value =* 2.2e-11) and SKCM (*P-value =* 3.7e-18) according to the correlation analysis (26). These findings support the idea that *NRP1* and *NRP2* in the TME are enriched in Type 2 TAM that adapt to the microenvironment-derived signals influencing disease progression.

In order to show that macrophage polarisation is context-dependent and linked to the TME modulation in which *NRP1* and *NRP2* are expressed at a higher level, leading to the state of M2-type TAM, further screening and identification of TAM-genes were conducted.

### Screening and identification of differentially expressed genes in TAMs with *NRP1* and *NRP2* in ccRCC and SKCM

*NRP* transcript variant expression in TAM is cancer type-specific. Different gene-sets are identified with the increased expressions of specific NRP type, as can be seen by differentially expressed genes (DEG). This suggests that TAMs with differential NRP transcript variants and related specific gene sets modulate the TME.

In the ccRCC dataset, macrophage cell isolation yielded 25,728 distinct gene features across 3,676 samples. Equally, a comprehensive transcriptional profile was obtained for the SKCM dataset, encompassing 22,846 unique gene features across 115 macrophage cells. Following the identification of the expression levels of *NRP1* and *NRP2*, the macrophages were classified into two heterogenous populations (*NRP^+^ or NRP^-^)* for each condition through PCA.

Subsequently, differential expression gene analysis (DEGA) identified a list of genes that were highly expressed in one group but not in the other, clearly distinguishing the genes associated with one of the NRP isoforms.

### Heterogeneity in TAM populations highlight spatial and functional variability in NRP1 and NRP2 expressions

**Figure 5:**
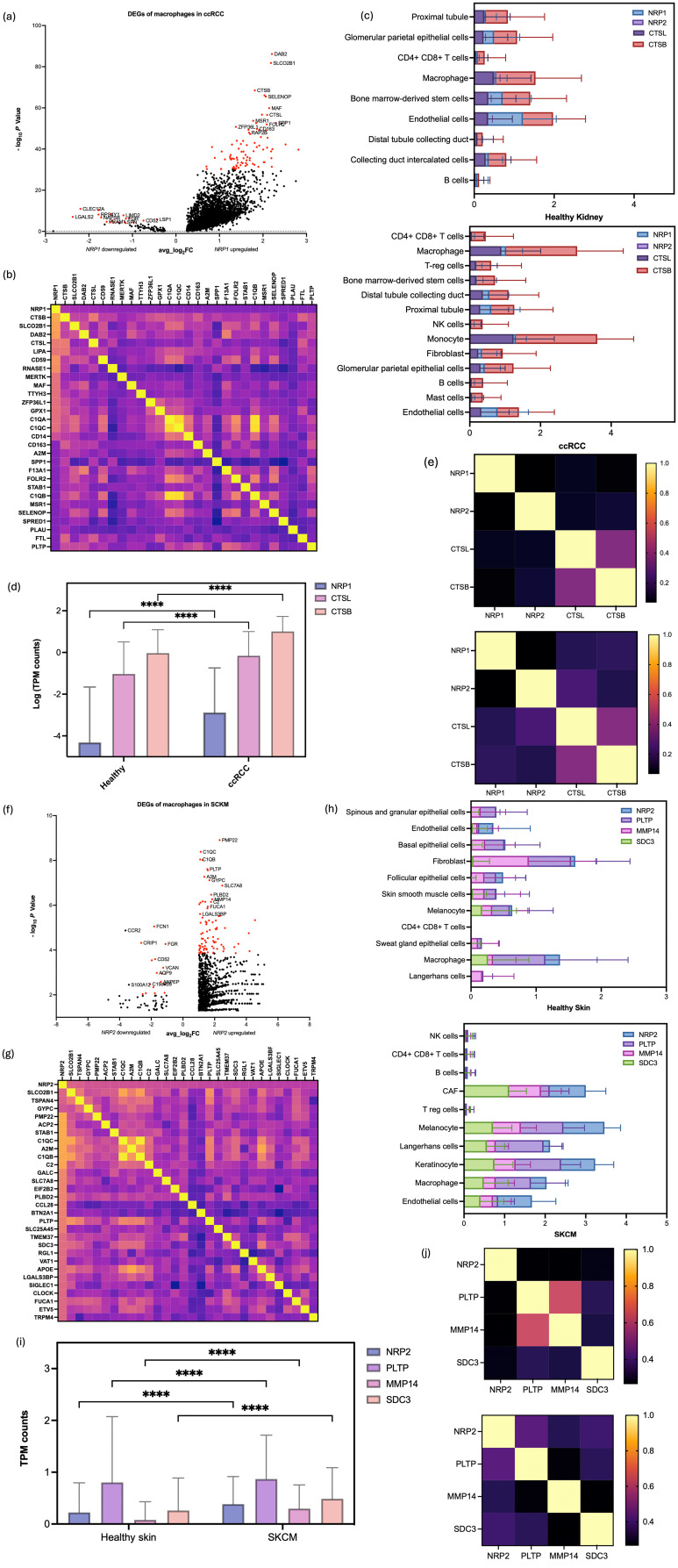
Multifaceted Gene Expression Analysis revealing NRP-related DEGs in the extracted macrophages. **(a)** Volcano plot to show the DEGs outlined for each of the NRP1^+^ and NRP1^-^ subgroups with the PCA results differentiating the heterogenous groups of cells based on the presence of NRP1, with P values and average Log_2_FC values in ccRCC samples. Top 100 genes from each subgroup were selected for the downstream analysis based on their P value in ascending order, provided they met the criteria of P < 0.05 and an average log fold change (average log2FC) above 1.0, indicating statistical significance. **(b)** Heatmap of the correlation matrix between the DEGs to identify NRP1+ -related genes across the macrophage subsets in ccRCC. **(c)** Quantitative analysis of NRP1, NRP2, CTSB, and CTSL expression across various cell types, comparing healthy kidney and ccRCC samples, reveals a significant increase in gene expression within the tumor environment. **(d)** TPM gene counts for NRP1, CTSL, and CTSB in macrophages highlight a significant upregulation in the ccRCC samples compared to healthy kidney samples. **(e)** Correlation plots indicate a stronger correlation between CTSL, CTSB, and NRP1 in ccRCC samples (right panel) than in healthy kidney samples (left panel), suggesting a potential role in tumorigenesis. **(f)** Volcano plot to show the DEGs outlined for each of the NRP2^+^ and NRP2^-^ subgroups with the PCA results differentiating the heterogenous groups of cells based on the presence of NRP2, with P values and average Log2FC values in SKCM samples. **(g)** Heatmap of the correlation matrix for NRP2^+^ -related genes across the macrophage subsets in SKCM. **(h)** Quantitative analysis of NRP2, PLTP, MMP14, and SDC3 expression across various cell types compares healthy skin and SKCM samples, demonstrating significant gene upregulation in the tumor environment. **(i)** TPM gene counts for NRP2, PLTP, MMP14, and SDC3 in macrophages reveal a marked increase in SKCM samples compared to healthy skin samples. **(j)** Correlation plots show an altered relationship between PLTP, MMP14, SDC3, and NRP2 in the tumour microenvironment of SKCM, contrasting with the healthy skin samples. ****p<0.0001.

Amongst the macrophage cell populations, DEGs were identified from the *NRP1^+^* and *NRP1^-^* from the ccRCC sample (**Figure 5a**), and *NRP2^+^* and *NRP2^-^* groups from the SKCM sample (**Figure 5f**). Genes that show a marked difference in expression between the two groups may be functionally related to the activity of *NRP1* or *NRP2* or their functions influencing cellular states.

While the DEGs identified from the *NRP-*negative groups were directly processed for the functional analysis, multigene correlation analysis was performed from the list of DEGs to find a cohort of genes directly associated with *NRP1* and *NRP2* expression in macrophages of the ccRCC and SKCM samples, respectively (**Supplementary Tables S6**, **S7**).

The correlation matrix heatmap showcased patterns of co*-*expression, suggesting potential regulatory networks (**Figures 5b**, **5g**). Notably, a cluster of genes showed positive correlation coefficients, delineating a putative gene expression signature linked to *NRP1* and *NRP2* function in the ccRCC and SKCM macrophage subset, respectively.

The expressions of *CTSB* and *CTSL*, genes highlighted from DEGA, were examined at the single cell level in both healthy and ccRCC samples (**Supplementary Figure S8**). Their expressions were notably increased not only amongst the macrophages, but also across the different cell types in the tumour (**Figure 5c**). This suggests that although *NRP1* might have a TAM-specific role in ccRCC, *CTSB* and *CTSL* might also function more broadly across multiple cell types in the TME. Especially TAMs showed significantly higher TPM counts of *CTSB* and *CTSL* compared to the healthy sample (P value < 0.0001) (**Figure 5d**). The broad expression of *CTSB* and *CTSL* in other tissues and cell types aligns with their general role in proteolytic pathways, but their co-expression with *NRP1* in macrophages hints at a cancer-specific regulatory network. *CTSL* and *CTSB* which hardly presented correlations with *NRP1* and *NRP2*, respectively, in the healthy tissue started to show correlations in the ccRCC sample, suggesting a potential mechanistic link involving *NRP1* in the ccRCC pathogenesis (**Figure 5e**).

For SKCM, the expression levels of *PLTP, MMP14*, and *SDC3*, which were low in healthy skin, were significantly elevated in SKCM, particularly within specific cell clusters, mirroring *NRP2* expression patterns (**Supplementary Figure S8**). While adaptive immune cells showed decreased expressions in SKCM, cancer-associated fibroblasts, melanocytes, Langerhans cells, keratinocytes, and endothelial cells exhibited notable increases of those DEGs, along with macrophages (**Figure 5h**). Specifically, *NRP2, PLTP, MMP14*, and *SDC3* showed significantly elevated TPM counts in TAMs of SKCM compared to healthy skin macrophages (P < 0.0001) (**Figure 5i**). Interestingly, gene correlation analyses revealed significant alterations in the TME: *PLTP* and *MMP14*, which were highly correlated in healthy skin, showed negligible correlation in SKCM. The loss of correlation reflects the broader phenomenon of tumour-driven functional reprogramming. TME components that were interdependent in healthy tissue may become independent or even antagonistic as tumours evolve. Conversely, *PLTP, MMP14*, and *SDC3*, which had weak correlations with *NRP2* in the healthy skin, exhibited strong correlations in SKCM (**Figure 5j**). The described shift in correlation between *PLTP* and *MMP14* highlights how tumour progression can fundamentally rewire cellular interactions and molecular pathways within the TME. These findings suggest that the transition to the TME influences gene expression and interaction with *NRP1* and *NRP2*, potentially contributing to the distinct mechanisms underlying ccRCC and SKCM pathogenesis, which could be the driving force of the cancer type-specific involvement of the NRP isoforms.

### Differently modulated TME as a result of TAM involving different gene-sets with NRP isoforms

To understand the main functions of the rigorously selected 47 *NRP1-*related DEGs and 91 *NRP2-*related DEGs upregulated in macrophages from the ccRCC and SKCM samples (threshold > 0.5), Gene Ontology (GO) and Kyoto Encyclopedia of Genes and Genomes (KEGG) enrichment analyses on the DEGs were performed. These analyses revealed distinct functional profiles, underscoring the heterogeneity and specialized roles of TAM subpopulations within the tumor microenvironment (TME)

for each of the *NRP1-*high and *NRP2-*high ccRCC and SKCM cases.

**Figure 6:**
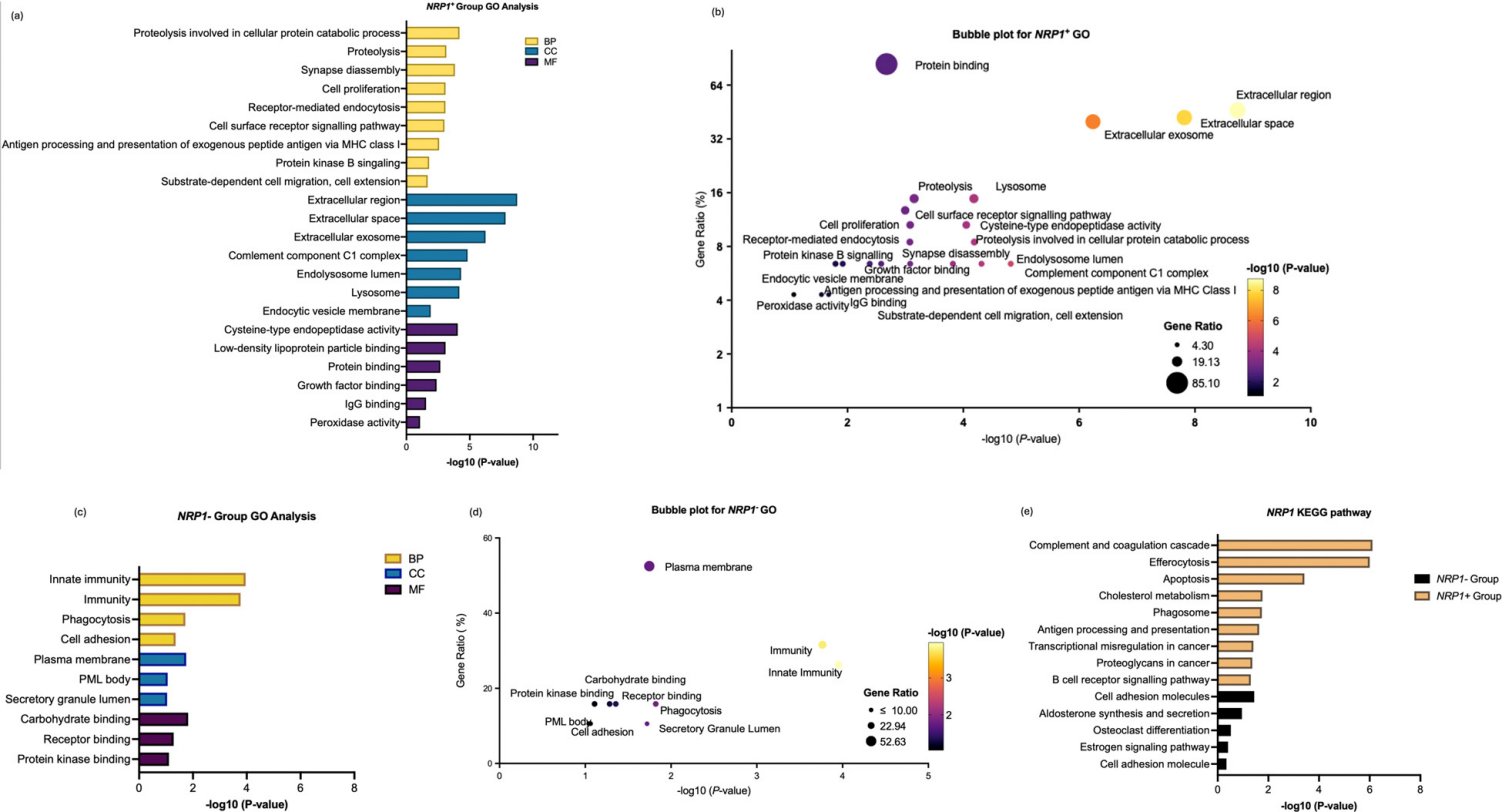
Enrichment analysis of differentially expressed genes between *NRP1* ^+^ group and *NRP1* ^-^ group in ccRCC. **(a)** GO enrichment analysis of differentially expressed genes highly correlated with NRP1 within the NRP1^+^ group across biological processes (BP), cellular compartments (CC), and molecular functions (MF). **(b)** Bubble plot to show the gene ratio and significance of the GO terms of the genes in the NRP1^+^ group. **(c)** GO enrichment analysis of differentially expressed genes in NRP1^-^ group. **(d)** Bubble plot to show the gene ratio and significance of the GO terms of the genes in the NRP1^-^ group. **(e)** KEGG enrichment analysis of differentially expressed genes in both *NRP1* ^+^ and NRP1^-^ groups in ccRCC. (Software: R (4.0.2) version, R packet: clusterProfiler (4.6.2). URL: https://bioconductor.org/packages/release/bioc/html/clusterProfiler.html) and DAVID programme server (https://david.ncifcrf.gov/webservice/services/DAVIDWebService).

In the *NRP1* ^+^ group, GO enrichment analysis across BP, CC, and MF highlights their active engagement in extracellular matrix remodeling and lysosome-dependent antigen processing. The enrichment of growth factor-binding functions also underscores the involvement of *NRP1* ^+^ macrophages in VEGF-mediated angiogenesis, a hallmark of ccRCC. Notably, processes such as substrate-dependent cell migration and cell extension align with the well-documented role of TAMs in facilitating tumour invasion and metastasis. Interestingly, cholesterol metabolism was also highlighted, which plays a key role in regulating macrophage functions through the use of lipids as a source of energy for TAMs and regulation of signal transduction during macrophage activation (**Figures 6a**, **6b**). By contrast, *NRP1* ^−^ macrophages exhibited more restricted functional enrichment. The *NRP1*− group demonstrated significant enrichment in plasma membrane and receptor-binding functions, further suggesting its involvement in more innate immune functions rather than pro-tumorigenic activities (**Figures 6c**, **6d**).

Interestingly, by carefully analysing the expression pattern of genes through DAVID and KEGG which enabled identification of the functions of *NRP1-*related gene-sets, the contrast *NRP^+^* TAMs showed against *NRP^-^* TAMs which lacked significant enrichment in most tumour-promoting pathways further highlighted the functional polarisation of *NRP1^+^* TAMs. Given that the gene-set exhibits a highly specialised pro-tumorigenic profile characterised, *NRP1* ^+^ TAMs are expected for matrix modelling, angiogenesis, and immune suppression. However, *NRP1* ^−^ macrophages appear to maintain a more pro-inflammatory, anti-tumour phenotype (**Figure 6e**).

**Figure 7:**
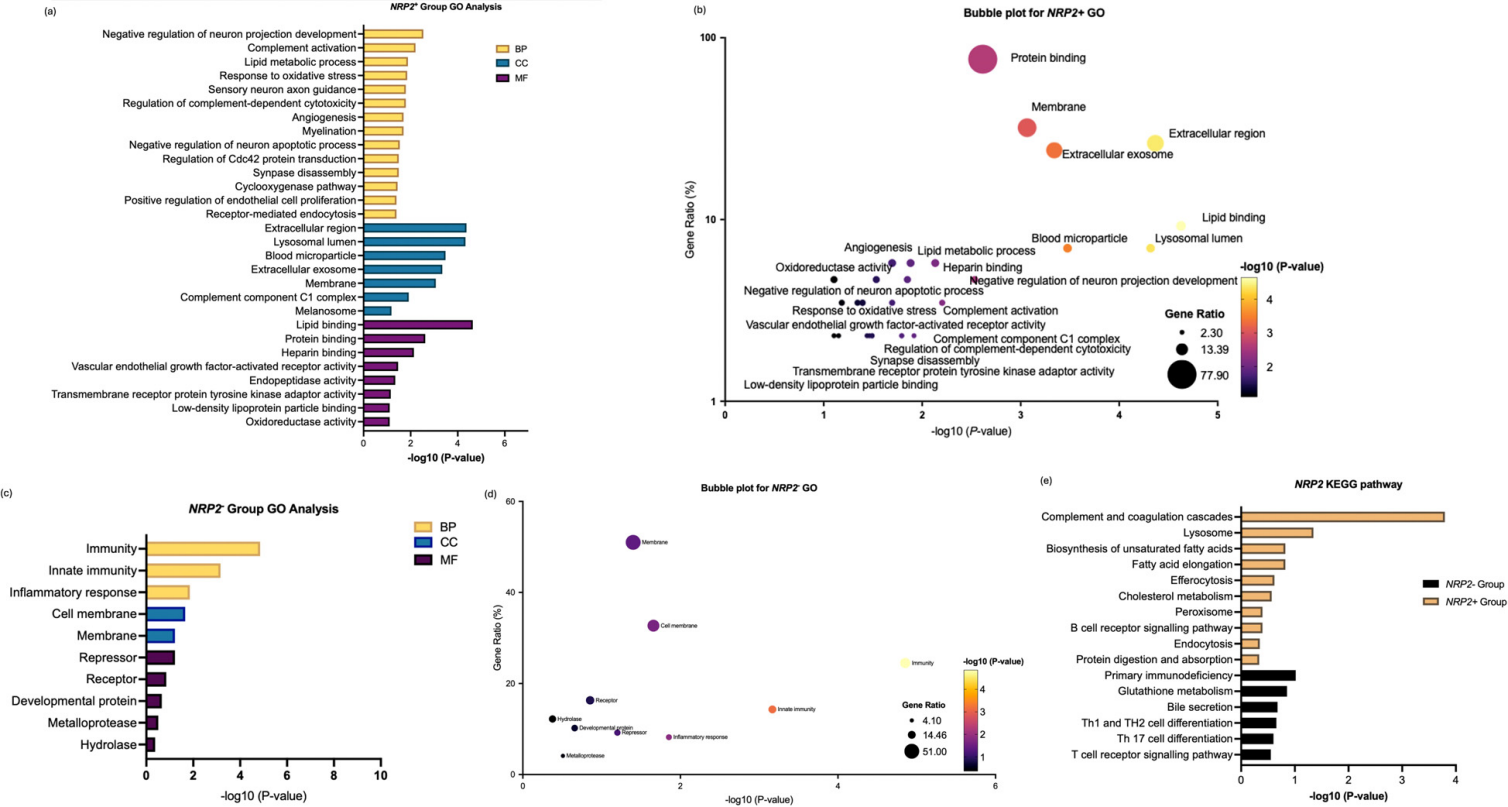
Enrichment analysis of differentially expressed genes between NRP2^+^ group and NRP2^-^ group in SKCM. **(a)** GO enrichment analysis of differentially expressed genes highly correlated with NRP2 within the NRP2^+^ group across biological processes (BP), cellular compartments (CC), and molecular functions (MF). **(b)** Bubble plot to show the gene ratio and significance of the GO terms of the genes in the NRP2^+^ group. **(c)** GO enrichment analysis of differentially expressed genes in NRP2^-^ group. **(d)** Bubble plot to show the gene ratio and significance of the GO terms of the genes in the NRP2^-^ group. **(e)** KEGG enrichment analysis of differentially expressed genes in both NRP2^+^ and NRP2^-^ groups in SKCM. (Software: R (4.0.2) version, R packet: clusterProfiler (4.6.2). URL: https://bioconductor.org/packages/release/bioc/html/clusterProfiler.html) and DAVID programme server (https://david.ncifcrf.gov/webservice/services/DAVIDWebService).

Enrichment analysis for *NRP2^+^* in **Figure 7** highlights the contribution of *NRP2* ^+^ TAMs to metabolic reprogramming and immune modulation within the TME. Unlike *NRP1^+^* TAM, *NRP2^+^* TAM shows more dual- and context-dependent roles of macrophages where they are enriched in both the pro-tumoral and anti-tumoral features. However, *NRP2^-^* TAMs focused on immune functions and had more of anti-tumouric characteristics by being more involved as an immune cell (**Figures 7a**, **7b**). On the other hand, genes in the *NRP2* ^-^ group showed distinct pathways compared to *NRP2* ^+^ TAM, highlighting processes less associated with pro-tumorigenic TAM activities, namely as anti-tumorigenic immune cells (**Figures 7c**, **7d**).

Comparative analysis revealed that *NRP1* ^+^ TAMs in ccRCC were predominantly engaged in lysosome-dependent antigen processing, ECM degradation, and VEGF-mediated angiogenesis, while NRP2+ TAMs in SKCM played a more prominent role in lipid metabolism, immune suppression, and neural-like environment remodelling. The functional divergence between *NRP1* ^+^ and *NRP2* ^+^ TAMs reflects the cancer type-specific heterogeneity and highlights their specialized roles in modulating the TME.

## Discussion

Through the differential isoform expression patterns of neuropilin at the mRNA level across different cancer types following their over-expression after tumorigenesis, cancer heterogeneity and disparately shaped TME has been highlighted. In particular, ccRCC samples showed the highest expression level of *NRP1* and SKCM samples revealed the highest level of *NRP2*, where each of them was expressed at a significantly higher level compared to the other *NRP* transcript variant. Interestingly, the expression levels of *NRP1* in ccRCC and that of *NRP2* in SKCM were distinctively elevated in macrophages compared to the healthy samples.

Macrophages in the TME exhibit dual functionalities - M1-like macrophages promote anti-tumour immune responses, while M2-like macrophages are known to drive tumour progression through angiogenesis, immune suppression, and extracellular matrix modelling (3, 4). Markers for the activated M2-like TAMs including *CD38, GPR18, FPR2, CD163, and EGR2* were upregulated amongst the macrophages of the ccRCC and SKCM. *NRP*s upregulated in cancer are highly expressed in macrophages, which show tendency towards the phenotypes of M2-type TAMs, suggesting that *NRP1* and *NRP2* could be more expressed in M2-type TAMs in ccRCC and SKCM, which could be the specific potential target.

According to the analysis of enriched genes in *NRP1^+^* TAM, *CTSL* and *CTSB* were identified, as part of the gene-set for *NRP1^+^* TAM. Cathepsin L, encoded by the gene *CTSL*, is capable of proteolytically processing CDP/Cux, leading to the generation of the physiologically significant p110 isoform, which exhibits a stable binding affinity towards the secreted VEGF, thereby facilitating the transcriptional upregulation of *VEGF* (27). In cancers, TAMs produce VEGF-A in hypoxic areas of tumours. VEGF, upon binding, interacts with the tyrosine kinase receptors (TKRs), VEGFR-2, which interact with NRP as a co-receptor (28). NRP1-b1 domain binding pocket structure is well-known for the interaction site for VEGF-A_165_ (29). With an enhanced level of VEGF secretion and upregulation of *NRP1*, such molecular cascade would contribute significantly to the angiogenic processes within cancer cells (30).

In addition, cathepsin B is encoded by *CTSB* functions both as an endopeptidase and exopeptidase, and the latter is notably associated with its pathological role in ECM degradation. Secreted *CTSB* has been demonstrated to aid invasion through ECM degradation by targeting structural components such as collagen and elastin for degradation (31). *CTSB* also acts indirectly by degradation of MMP inhibitors TIMP1 and TIMP2 leading to increased ECM degradation and angiogenesis (32). Additionally, the presence of CTSB on the surface of cells in contact with tumour cells facilitates the apoptosis of CD8 + T lymphocytes and the degradation of cytotoxic effector molecules (33–35). High vascularisation comes with a high level of immune cell infiltration in ccRCC (36). *NRP1* ^+^ TAMs may contribute to this through CTSB mediated CD8+ T-lymphocyte apoptosis, as well as more generally through their M2-like polarisation and associated immune suppressive signalling. The co-enrichment of *CTSL* and *CTSB*, in conjunction with elevated *NRP1* levels, contributes significantly to angiogenesis and tumor invasion, marking the progression from a healthy state to the development of ccRCC.

In SKCM, *NRP2^+^* macrophages presented different associated gene-sets and related pathways. *NRP2*, known for its association with lymphangiogenesis in cancer development, is here reported to correlate with *MMP14. MMP14* activates *MMP2*, potentially contributing to tumour invasion, as the lymphatic system serves as a significant route for tumour dissemination (37). Notably, enhanced membrane-bound MMP14 expression promotes cancer cell invasion via cdc42 activation. GO terms such as ‘regulation of cdc42 protein transduction’ and ‘cyclooxygenase’ were reported for the genes enriched in the *NRP2^+^* TAMs, which were not shown in the TAMs of ccRCC. Recently, knockdown of *Cdc42* led to an inhibition of migration and proliferation of cancer cells in TME in a paradoxical manner, due to the effect of *Cdc42* overexpression which can enhance the production of lactic acid and promotion into the polarisation of M2 macrophages which inhibit the function of T-lymphocytes. This may lead to tumour cell proliferation and migration through the PTEN/AKT pathway (38). Additionally, cyclooxygenase-2 (COX-2) in TAMs induces MMP-9 expression, further promoting EMT in cancer cells (39). This evidence provides a feasible mechanism explaining *MMP*-9 downregulation in NRP2-depleted macrophages (40).

In addition, *PLTP* is the gene for phospholipid transfer protein which is a member of the lipid transfer/lipopolysaccharide-binding protein family. *NRP2* and *PLTP* collectively enhance ECM remodelling, facilitating tumour invasion, as *PLTP* could supply necessary lipids for protease activity or membrane dynamics involved in invasion. Notably, *PLTP*-expressing *C1QC*-positive TAMs have been shown to modulate the abundance of dysfunctional T-lymphocytes through cytokine and chemokine signalling in the context of metastasis within the TME (41).

Furthermore, syndecan-3 encoded by *SDC3* is known to regulate cell adhesion, and is involved in ERK and AKT signalling (42). The complexity of *NRP2* involvement in SKCM is associated with its co-enrichment with *SDC3*. It has been shown that *SDC3* expression in melanoma TAMs is positively regulated by HIF1α (43), which has also been shown to repress NRP2 promoter activity and expression in another cancer type, lung adenocarcinoma (44). This apparent contradiction in *NRP2* co-enrichment with *SDC3* highlights the complexity and context-dependence of control over NRP1/2 isoform expression, suggesting the involvement of other factors besides hypoxic signalling. Further work is needed to understand what mediates the molecular switch of NRP1/2 expression in TAMs.

However, GO and KEGG studies indicate both pro-tumoral and anti-tumoral characteristics of the genes enriched in *NRP2^+^* TAMs, which suggests that *NRP2* in SKCM could be based on a dual role, which can be context-based in the TME, which requires more considerations for future studies. Upregulation of *NRP2* during M2-like polarization and efferocytosis shows the complexity of the roles TAMs may play, as tumours can harness efferocytosis to prevent DAMP-induced inflammation with the high burden of apoptotic cells within a tumour, and phagocytosis itself leads to anti-inflammatory signalling through TGF-β and IL-10 secretion from the phagocyte (45). If *NRP2* is deleted, efferocytotic clearance of apoptotic tumour cells is impaired and secondary necrosis leads to activation of a more effective anti-tumour immune response (40), highlighting the importance of tumour modulation of the NRP molecular switch in TAMs for disease progression. However, downregulation of NRP2 suggests that isoform-specific roles, tissue-specific regulation, or post-translational modifications might underlie the dual functionality. Notably, polysialylation of NRP2 has been implicated in regulating cell migration and phagocytosis (46). It is plausible that polysialylated NRP2 functions as a molecular switch in TAMs, modulating their migration and capacity for efferocytosis in the TME.

Our findings align with the notion that NRPs serve as molecular switches that govern TAM behaviour in the TME, and that the regulation of this switch may involve HIF signalling. NRP1 and NRP2 each have distinct pathways they interact with which are relevant in the context of the cancers they are associated with, angiogenesis and lymphangiogenesis respectively. This divergence in function of NRP isoforms aligns with the structural differences in the b2 domains of NRP1 and NRP2, which interact with different ligands and binding partners. However, these results highlight how they overlap in their contribution to ECM remodelling and immune suppression in the TME. This adds to the growing body of evidence supporting the roles of M2-polarised macrophages in tumour progression, highlighting the NRPs, yet another way in which these effects are mediated, especially in the context of ccRCC and SKCM.

Those findings were also supported from other cell types including the Freshly isolated human peripheral blood mononuclear cells (PBMCs) and murine bone marrow-derived macrophages (BMDMs). PMBCs and MBDMs which have been demonstrated to express low basal levels of NRP2 showed markedly upregulated expressions upon activation with conditioned media derived from UNKC-6141 pancreatic ductal adenocarcinoma (PDAC) cells (40, 47). Similarly, PBMCs isolated from colorectal cancer patients exhibited low NRP1 expression compared to TILs. When co-cultured with tumor tissue from human colorectal cancer liver metastases, these macrophages showed induced NRP1 expression (47). Interestingly, Roy et al. demonstrated that 94.9% of BMDMs activated with conditioned media from a PDAC model cell line expressed CD163, a well-established marker of M2 macrophage polarization, with the majority of these cells also expressing NRP2 (7). Dhupar et al. further showed that TAMs with high NRP2 expression, isolated from 4T1-derived murine breast cancer models, co-expressed both CD86 and CD206, whereas TAMs with low NRP2 expression were predominantly associated with high CD86 expression only (48). Since CD206 is a canonical marker of M2 macrophages and CD86 is linked to T-cell activation and anti-tumorigenic activity, this suggests functional heterogeneity within TAM populations. Furthermore, TAMs with high NRP2 expression exhibited elevated levels of arginase-1 and inducible nitric oxide synthase (iNOS), markers associated with immune suppression and angiogenesis, respectively (48).

Ji et al. reported that monocyte differentiation with macrophage colony-stimulating factor (M-CSF) under conditions promoting an M2 phenotype significantly increased the expression of both NRP1 and NRP2, implicating these receptors in alternative macrophage activation independent of the TME. Conversely, stimulation of monocytes to an M1 phenotype with interferon-gamma (IFN-γ) reduced *NRP1* expression, while lipopolysaccharide (LPS) stimulation decreased *NRP2* expression. These findings further emphasize the complexity of signaling pathways regulating differential NRP isoform expression. Additionally, pharmacological and genetic ablation of NRP1 in macrophages and microglia induced anti-tumorigenic phenotypes in a glioma model, correlating with classical M1 macrophage activation (19, 49). Collectively, these data demonstrate that *NRP1* and *NRP2* expression is tightly linked to the M2 phenotype in TAMs, and that reducing their expression disrupts this pro-tumorigenic polarization.

This complexity underscores the need for precision therapies targeting specific NRP isoforms. The therapeutic implications of targeting NRPs in TAMs are significant. Pan-NRP inhibition could indiscriminately affect multiple pathways, emphasizing the importance of isoform-specific strategies (57). Additionally, the identification of gene sets co-expressed with NRP isoforms offers potential biomarkers for cancer diagnosis and prognosis, as well as therapeutic targets to reprogram TAMs and disrupt their pro-tumorigenic functions (59). The promise of this approach is clear as pharmacological inhibition of NRP1 not only reduces TAM recruitment but also reprograms their polarization toward an anti-tumorigenic M1-like state (49).

Beyond their roles in the TME, NRPs are increasingly recognized as regulators of systemic inflammation and metabolic dysfunction. For example, NRP1 has been implicated in macrophage activation during sepsis and in promoting inflammation in obesity-related metabolic diseases. Similarly, NRP2’s pro-reparative roles in resolving inflammation and promoting efferocytosis suggest its potential as a therapeutic target for chronic inflammatory disorders. These insights position NRPs as key regulators not only in cancer but also in broader contexts of immune and metabolic diseases. Targeting NRP isoforms and their associated gene networks offers a promising avenue for developing precision therapies that modulate TAM behavior and disrupt tumor progression. Future studies should explore the isoform-specific mechanisms and therapeutic potential of NRPs in diverse cancer types to refine strategies for immunomodulation and TME reprogramming through biological validations.

## Study limitations

Single-cell RNA sequencing outcomes generally show limitations in data sparsity and tissue dissociation biases. The latter is particularly relevant to adhesive cells, such as epithelial or tumour-programmed (TP) cell clusters. Thus, scRNA-seq datasets and the resulting cellular composition of tumours (as well as immune cells) can be influenced by the dissociation protocols and other experimental variables, inflating the immune compartment at the expense of the tumour cell capture. Whilst our efforts led to a finding of an association between the gene-sets and differential *NRP* isoforms in ccRCC and SKCM at the *in-silico* level, future studies are required to validate our findings. Moreover, functional *in-vitro* and *in-vivo* characterisation will be necessary to elucidate the role of *NRP* in the polarisation of macrophages into Type 2-like TAM. Another limitation inherent to the chosen study design pertains to compromises in the quality of scRNA-seq data and limited patient samples to conclude our findings.

Nevertheless, despite these constraints, our study identifies previously under-characterized cell populations and their potential interactions. In doing so, it not only complements the differential expression analysis of neuropilin isoforms but also highlights new avenues for future research.

## Conclusion

This study underscores the distinct and context-dependent roles of NRP isoforms, NRP1 and NRP2, in tumor-associated macrophages across different cancer types. NRP1-mediated angiogenesis in ccRCC and NRP2-driven lymphangiogenesis and tumor invasion in SKCM demonstrate the complexity of TME dynamics, and their shared role in ECM remodelling and cancer immune evasion provides novel insights into effector mechanisms of M2-polarised macrophages in the TME. The differential expression of NRP isoforms in TAMs, and their associated enriched gene sets, highlights their potential as therapeutic targets for precision medicine. Future studies should explore isoform-specific mechanisms, post-translational modifications, the control mechanisms of the NRP1/2 molecular switch, and the broader implications of NRPs in immune and metabolic disorders. This study hopes to provide the basis for future studies through narrowing-down specific gene-sets to focus on. Targeting NRP isoforms offers a promising strategy to modulate TAM behavior and inhibit tumor progression, with the potential to improve cancer therapies and beyond.

## Materials and methods

### Comparison between *NRP1* and *NRP2* mRNA expression levels across different cancer types

32 TCGA datasets including 10,071 tumour samples were downloaded from cBioPortal​ (50, 51)​ to assess the expressions of *NRP1* and *NRP2* mRNA across 19 different cancer types. mRNA expression levels of *NRP1* and *NRP2* measured in RNA*-*Seq by Expectation Maximization (RSEM) were assessed. The datasets – batch*-*normalised from Illumina HiSeq_RNASeqV2 – were illustrated by two box and whisker plots constructed with turkey whiskers representing the medians and quartiles of *NRP1* and *NRP2* expressions across the 19 cancer types. One-way ANOVA with the follow-up test of Dunnett’s multiple comparisons test was conducted to compare the mean differences of RSEM values between the cancer types.

### *NRP1* and *NRP2* mRNA expressions for ccRCC and SKCM

RNA*-*Seq data from TCGA (https://www.cancer.gov/tcga) were downloaded and processed. To compare expression of the genes of interest in a tissue specific manner, quantile normalised RNA*^-^* Seq data were obtained from the UCSC Xena web server (http://xena.ucsc.edu). The RNA*^-^* Seq dataset comprised a comprehensive set of tumour samples from 10,535 samples of TCGA as well as healthy controls from Genotype*-*Tissue Expression Project (GTEx) for different tissues of origin. The normalised RSEM counts for ‘Primary Tumour’ and ‘Normal Tissue’ were selected from the sample type under the TCGA Target GTEx cohort within the primary sites, kidney and skin (n=1937). For the levels of mRNA expressions of *NRP1* and *NRP2* in ccRCC and SKCM, TCGA Pan*-*Cancer (PANCAN) cohort was selected (n=1079). The mRNA gene expression was reported following the normalisation of the RNA seq-batch effects, with the data log*^-^* scaled, expressing the data in log2(norm_value *+*1). The datasets were downloaded for all the available samples, and re*-*plotted. Welch’s t*-*test was used to show the significantly different expression levels of the *NRP* isoforms in each of the cancer types.

### Multiple gene comparison in ccRCC and SKCM matched with TCGA and GTEx data

Through GEPIA2 web server (http://gepia2.cancer*^-^* pku.cn), multiple gene comparison was performed under the GTEx and TCGA datasets for the selected genes highlighted through STRING v.11 (52) which showed relevance in the pathway to compare the differential expression levels in ccRCC and SKCM, also against the healthy samples. The function – ‘Multiple Genes Comparison’ under ‘Expression Analysis’ was used to plot an expression matrix plot based on the gene list generated by STRING, based on the median expression values of each gene in the kidney and skin, which was visualised by the density of colour in each block. The values were normalised by the maximum median expression value using log_2_(TPM*^+^* 1) across all blocks, which were compared in the same tumours and normal tissues through matching with the TCGA normal and GTEx data in one plot.

### Clustering of single cells and identification of *NRP1* and *NRP2* in the healthy kidney and ccRCC samples at the single cell level

sc-RNAseq data for healthy kidney cells was downloaded from a publicly published dataset (GSE131685) which was based on the study conducted by Liao et al. (PMID: 31896769) (53) to understand the differential levels of *NRP1* and *NRP2* expressions in the healthy kidneys based on the kidney specimens from organ donors (two males and one female) aged 57*-*65 years. The three different samples were separately downloaded, which were labelled as ‘k1’, ‘k2’, and ‘k3’ before the merge of the samples altogether before the downstream process (**Supplementary Figure S1**). To investigate differential *NRP1* and *NRP2* expressions in ccRCC patients at the single*-*cell level, we used a public sc-RNAseq dataset, downloaded from the dbGaP website, under phs002065.v1.p1 (20), in which cells from primary and metastatic sites of human ccRCC patients were annotated (20). 8 patients with advanced ccRCC were included in the dataset, with 25,728 genes across 38,342 cells after QC (**Supplementary Figure S1**). Analysis was conducted using the Seurat Package (54) on R Version 4.3.2 (55). Using Seurat for quality control (QC), the number of genes and number of UMI in each cell were calculated. During the quality control phase, we excluded cells of suboptimal quality and discarded empty droplets characterized by minimal gene expression. Additionally, cells with unique feature counts exceeding 2500 or falling below the 200*-*threshold, as well as those with mitochondrial counts surpassing 5%, underwent systematic filtration. Within this process, the quantification of unique genes within each cell served as a surrogate metric for both sequencing depth and overall cell quality. After QC, high*-*quality cells were maintained altogether for the down*-*stream analysis. A linear transformation (‘scaling’) was applied that is a standard pre-processing step prior to dimensional reduction techniques like PCA, in order to shift the expression of each gene, so that the mean expression across cell is 0, and the variance across cell is 1, to give equal weight in downstream analyses, to ensure that highly-expressed genes do not dominate. Dimensionality reduction and unsupervised clustering for the cells were achieved using the Principal Component Analysis (PCA) and t*-*distributed Stochastic Neighbour Embedding (tSNE) algorithm. The tSNE analysis was performed with a resolution of 0.4, as higher resolution values (above 0.6) caused over*^-^* clustering, with edges having a low proportion ratio and unstable clusters. To overcome the extensive technical noise in sc-RNAseq data, ‘ElbowPlot()’ function was employed to generate a ranking of principle components based on the percentage of variance explained by each one. Based on the ‘elbow’ values around PC 14-15, which suggests that the majority of true signal is captured in the first 14 PCs, the dimensionality were determined around this value. Same method was used for determining the dimensionality for the other datasets. tSNE visualisation based on origins showed that each cluster was derived from specific kidney or immune cells, thus each cluster was expected to have a specific gene profile. According to the gene signatures specified in the paper (20), each cluster was identified with the specific markers (**Supplementary Table S1**).

### Identification of the cell clusters associated with tumour programme for ccRCC and relevance for the different *NRP* isoform

According to the gene markers identified as malignant highlighted by Bi et al. (20), cells showing cancer*-*specific genes were subclustered as tumour programme (TP) based on the established clusters. Here, TP referred to cellular programmes active in cancer cells which may drive interactions with the immune system by showing shared patterns of expression across cancer cells. Then the expression levels of *NRP1* and *NRP2* were confirmed in those subclusters of TP1 and TP2 to indicate the differential expression levels of *NRP1* and *NRP2* in the clusters directly associated with tumour progression through the ‘FeaturePlot’ function on R.

### Clustering of single cells in the healthy skin and SKCM patient samples and identification of the differential expressions of *NRP1* and *NRP2*

Recently published sc-RNAseq data by He et al. encompassing 84,363 cells obtained from various healthy tissues for an adult human cell atlas (21) was retrieved from the publicly accessible dataset, specifically focusing on the skin tissue data (GSM4850587). For the sc-RNAseq analysis of SKCM data, we downloaded a gene expression dataset which was publicly available on Mendeley, in which cells from primary and metastatic sites of human SKCM patients were annotated (22). 22,846 genes across 3,700 cells were obtained after QC from 4,646 cells (**Supplementary Figure S2**). The selection of diverse cell types was accomplished through the utilisation of cluster expression profiles comprising canonical markers associated with each cell type (**Supplementary Table S2**). Subsequent analysis was executed within the R environment (55), leveraging the Seurat Package (54). Within the framework of Seurat, the same QC measures were applied as for the kidney datasets. For each cell, the number of genes and the number of unique molecular identifiers (UMI) were meticulously quantified (**Supplementary Figure S2**). Dimensionality reduction and unsupervised clustering for the cells were achieved using the PCA and tSNE algorithm. The tSNE analysis was performed with a resolution of 0.5 to cluster the cells into groups of the same cell type, which was evaluated through the expressed gene markers. Subsequently, relative quantification of *NRP1* and *NRP2* expressions within the clustered cells of SKCM patients was obtained. These quantitative insights were visualised using violin plots dedicated to the presentation of *NRP1* and *NRP2* expression profiles.

### Expressions of *NRP1* and *NRP2* in SKCM TP cell clusters

For the identification of SKCM*-*specific ‘malignant’ genes representing TP, the TP*-*associated genes were subsequently marked on the tSNE map, which provided clarity regarding the clusters to which they belonged. Clusters showing pronounced expression levels of these TP genes were earmarked for further downstream analysis. Then the expression levels of *NRP1* and *NRP2* were confirmed in both TP and normal groups to indicate the differential expression levels of *NRP1* and *NRP2* which could be directly associated with tumour progression.

### Differentially expressed gene screening and identification of *NRP*-related genes

Macrophage cells were selected and isolated from the sc-RNAseq datasets of ccRCC and SKCM based on predetermined cell*^-^* type clustering parameters. The presence of NRP was used as a biomarker to categorise those macrophages, which enabled grouping into neuropilin*-*positive (*NRP1^+^* or *NRP2^+^*) or neuropilin*-*negative (*NRP1^-^* or *NRP2^-^*) based on the gene expression threshold set at zero. Subsequently, through PCA, macrophage cell populations with a presence of NRP and absence of NRP were classified, dividing the cells into two groups ‘NRP*^+^*’ and ‘NRP*^-^*’ for ccRCC and SKCM, respectively, Differential Gene Expression Analysis (DGEA) was performed to compare the *NRP1^+^* versus *NRP1^-^* groups within the ccRCC macrophage cells and *NRP2^+^* versus *NRP2^-^* groups within the SKCM macrophage cells. This analysis was aimed at identifying genes that were differentially expressed between the NRP*^+^* and NRP*^-^* groups, which could suggest distinct cellular functions associated with neuropilin expression.

Genes in the *NRP1^+^* and *NRP2^+^* groups demonstrating significant differential expression were subsequently analysed for correlation with neuropilin expression levels within ccRCC and SKCM, respectively. Top genes from each condition were subjected to a multigene correlation analysis against NRP (*NRP1* for ccRCC and *NRP2* for SKCM). The screening criteria of differentially expressed genes were | log2FC | > 1 and adjusted P value < 0.05, denoting statistical significance and substantial differential expression, respectively.

### Enrichment analysis of GO and KEGG enrichment analysis of *NRP* isoform-specific differentially expressed genes

Gene Ontology (GO) analysis and Kyoto Encyclopaedia of Genes and Genomes (KEGG) analysis of the ccRCC and SKCM datasets were performed using the “clusterProfiler” package in R46, together with DAVID programme server (56). In order to understand the roles of the differential NRP isoforms in the two different cancer types, DEGs specifically related to *NRP1* and *NRP2* as revealed in the upstream analysis were selectively included for the GO and KEGG enrichment analysis. GO analysis consists of three components: BP, CC, and MF. P < 0.05 was recognized as a significant term and pathway.

## Data Availability

The original contributions presented in the study are included in the article/**Supplementary Files**, further inquiries can be directed to the corresponding author/s.
