# Supplementary material for: Differential neuropilin isoform expressions highlight plasticity in macrophages in the heterogenous TME through in-silico profiling
Source: Front Immunol. 2025 Mar 11;16:1547330. doi: 10.3389/fimmu.2025.1547330 (PMC11933088; doi:10.3389/fimmu.2025.1547330)
Supplement: Supplementary file 1 [file DataSheet1.docx]

**Supplementary Files**

**Supplementary Figures**

**Figure S1.**

**
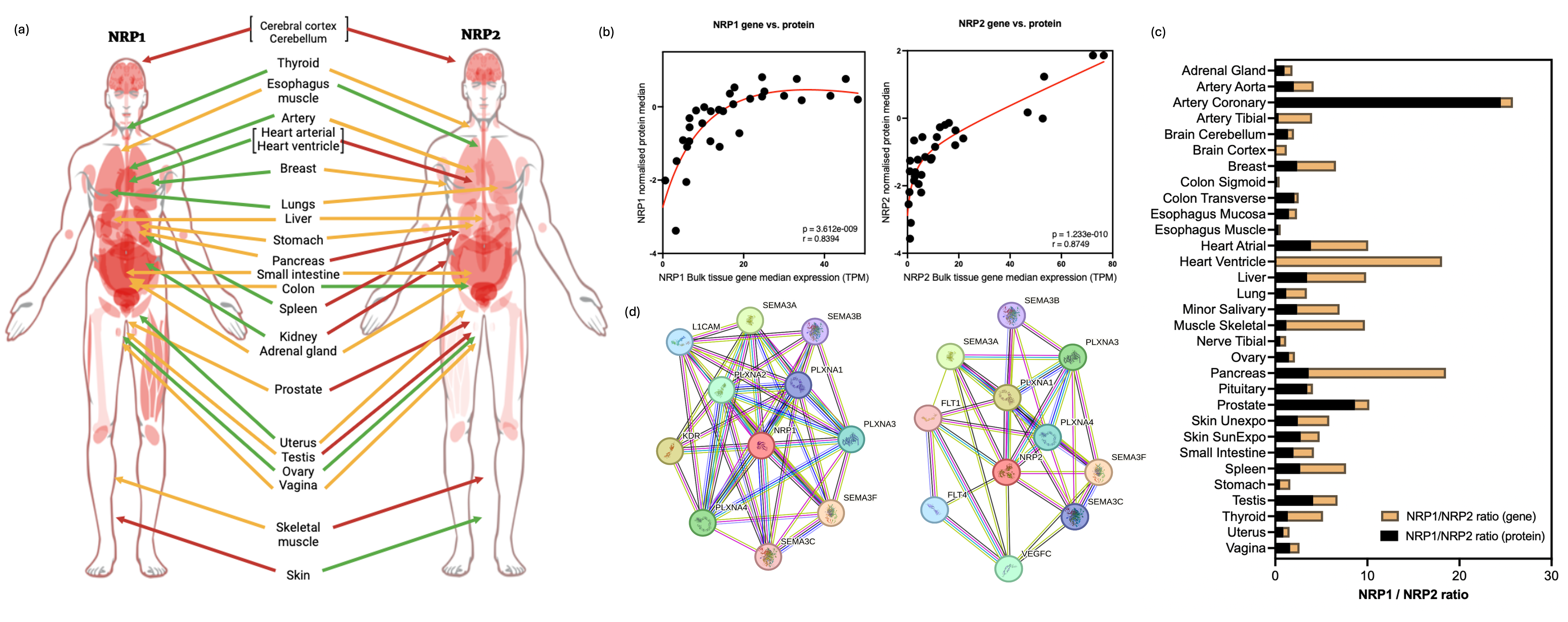
**

**Figure S1 Isoforms of NRP are differentially expressed and distributed at both gene and protein levels across the healthy human body.** (a) A quantitative proteome map for NRP1 and NRP2 distributions across various tissue types based on the median values of protein relative abundance. Green arrows refer to a high abundance with an estimated number of NRP identified above 0.1; yellow arrows refer to the median between 0.1 and -1.68; and red arrows refer to a low abundance with less than -1.68 NRP abundance identified. (b) Nonparametric Spearman correlation computed for NRP1 (left panel) and NRP2 (right panel) between the bulk tissue gene median expression (TPM) and normalised protein median with spearman r value and two-tailed P values calculated. Both NRP1 and NRP2 showed significant correlations between their gene expression levels and protein expression levels across the different tissue types. (c) The ratio between NRP1 and NRP2 across the different tissue types at the gene and protein levels. (d) Top 10 genes most closely related to and interacting with NRP1 and NRP2, respectively. The maximum number of interactors for the shell was no more than 10 interactors and the score of predicted functional partners was >0.995. NRP1, neuropilin 1; VEGFA, vascular endothelial growth factor A; KDR, kinase insert domain receptor; FLT1, FMS-related tyrosine kinase 1; SEMA3A, semaphorin 3A; PLXNA1, plexin A1, coreceptor for SEMA3A, SEMA3C, SEMA3F and SEMA6D; PLXNA2, plexin A2, coreceptor for SEMA3A and SEMA6A; PLXNA4, plexin A4, coreceptor for SEMA3A; SEMA3C, semaphorin 3C; SEMA3F, semaphorin 3F; phosphate, phosphoric acid; SEMA3D, semaphorin 3D; SEMA3E, semaphorin 3E; VEGFB, vascular endothelial growth factor B; PLXND1, plexin D1, and cell surface receptor for SEMA4A.

**Figure S2.**


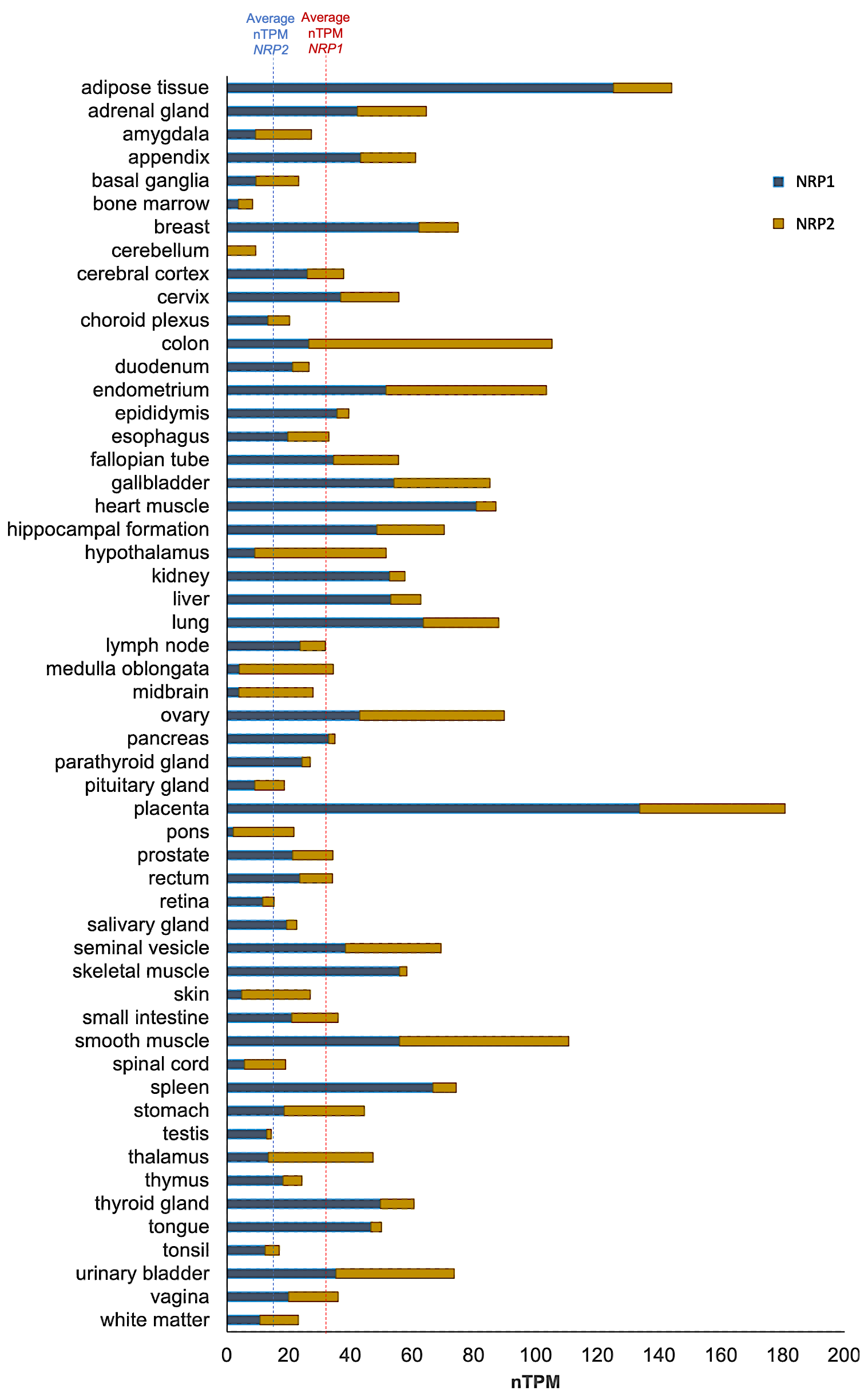


**Figure S2 Variance in the gene expression levels for the two different NRP isoforms amongst the different tissue types.** Distributions of NRP1 and NRP2 at the mRNA level standardised through the consensus normalised transcript expression (nTPM) values of NRP1 and NRP2 across 54 tissues.

**Figure S3.**


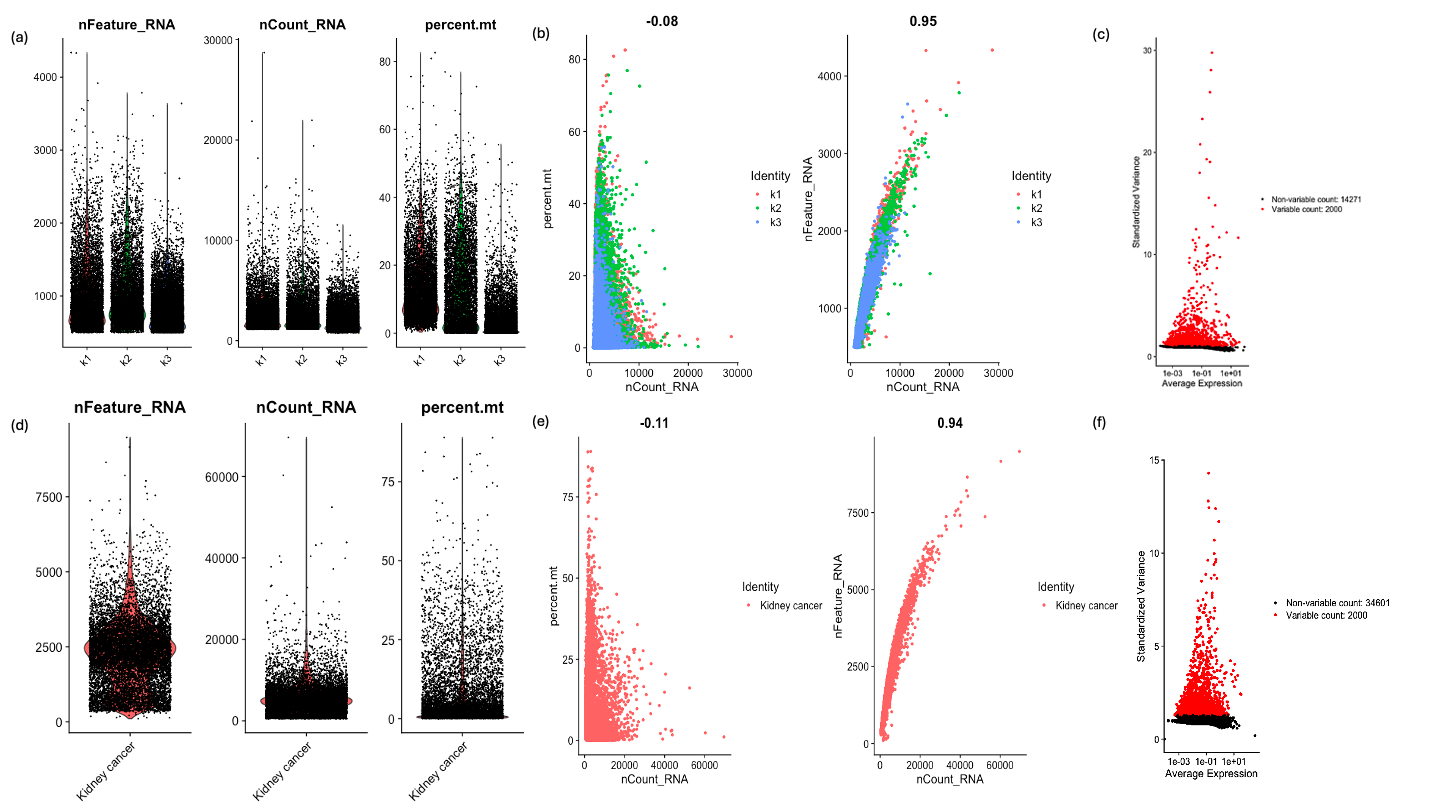


**Figure S3** **QC of healthy kidney and ccRCC samples before the downstream analyses.**  (a) Plots of quality control (QC) metrics which include the number of genes, number of unique molecular identifiers (UMIs), and the percentage of transcripts mapping to the mitochondrial genome. Violin plots of scRNA-seq data of healthy kidney sample (GSE131685). After QC, 23,366 high-quality cells were maintained altogether for the down-stream analysis, which was illustrated as 'nFeature_RNA' and 'nCount_RNA'. (b) Sample gene plots detecting cells with deviant levels of mitochondrial transcripts and UMIs post QC. (c) Subset of features exhibiting high cell-to-cell variation in the GSE131685 dataset was calculated by directly modelling the mean-variance relationship inherent in single-cell data, returning 2,000 features per dataset by default for PCA. (d) Violin plots of scRNA-seq data of ccRCC sample (phs002065.v1.p1). After QC, 26771 cells with 25728 features were obtained from the selected samples. (e) Sample gene plots detecting cells with deviant levels of mitochondrial transcripts and UMIs post QC. (f) Mean-variance relationship inherent in single-cell data to show the subset of features exhibiting high cell-to-cell variation in the phs002065.v1.p1dataset was calculated.

**Figure S4.**


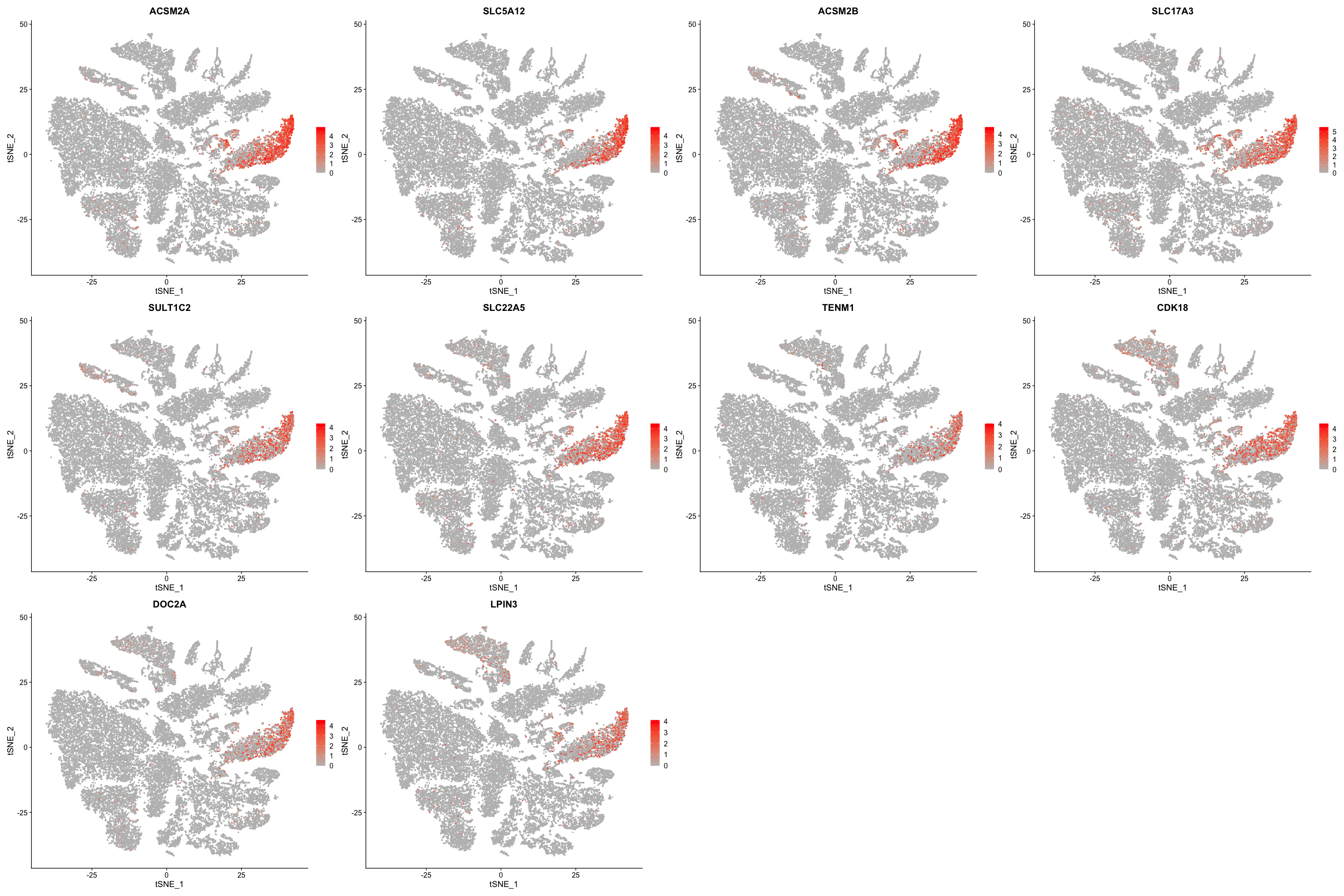

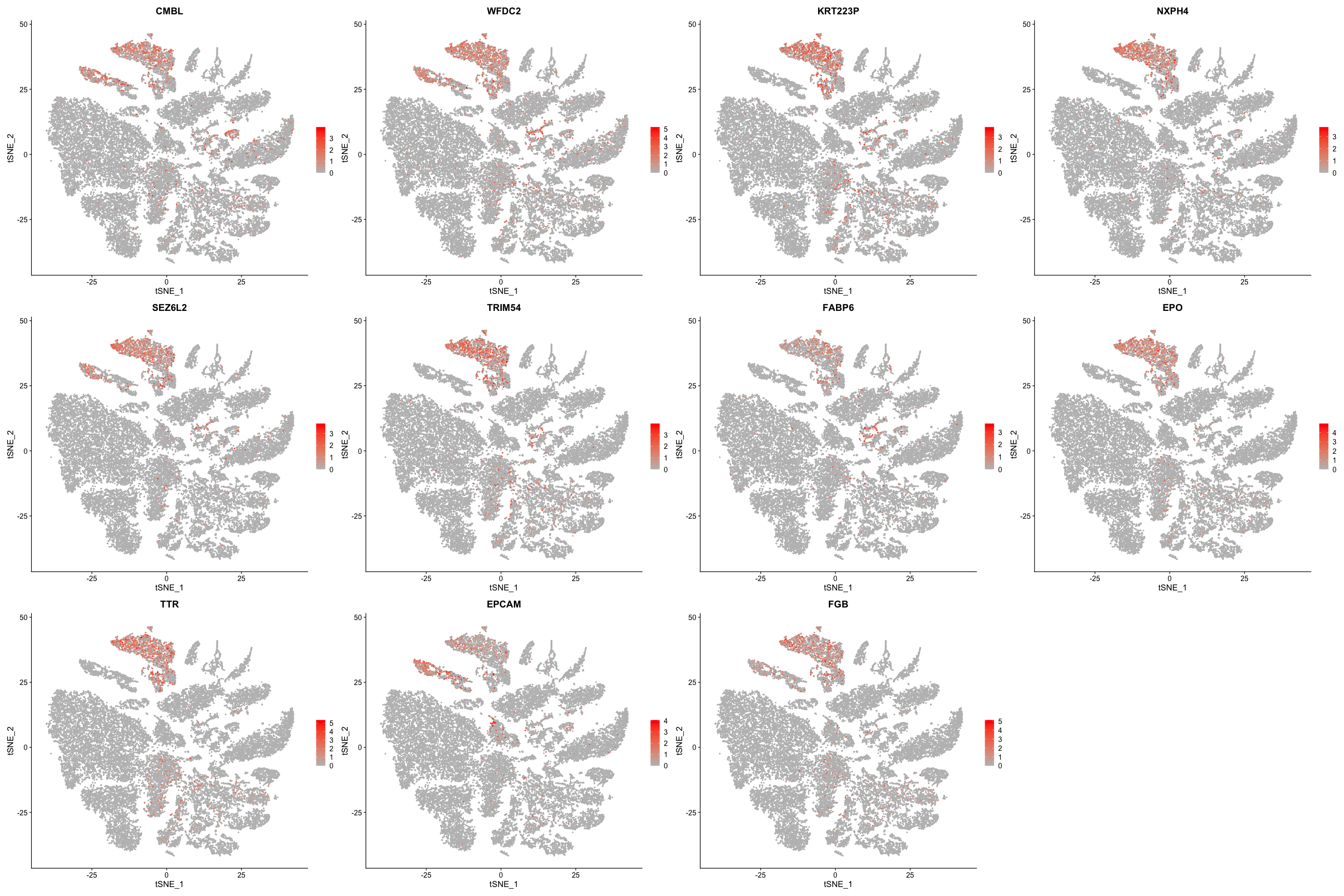


**Figure S4. Signature markers for tumour programmed genes in ccRCC marked in the tSNE plot plotted for the ccRCC analysis.** This shows which cluster group correlate with the genotypic features of TP. The markers used were based on the malignant gene expressions according to Bi et al.

**Figure S5.**


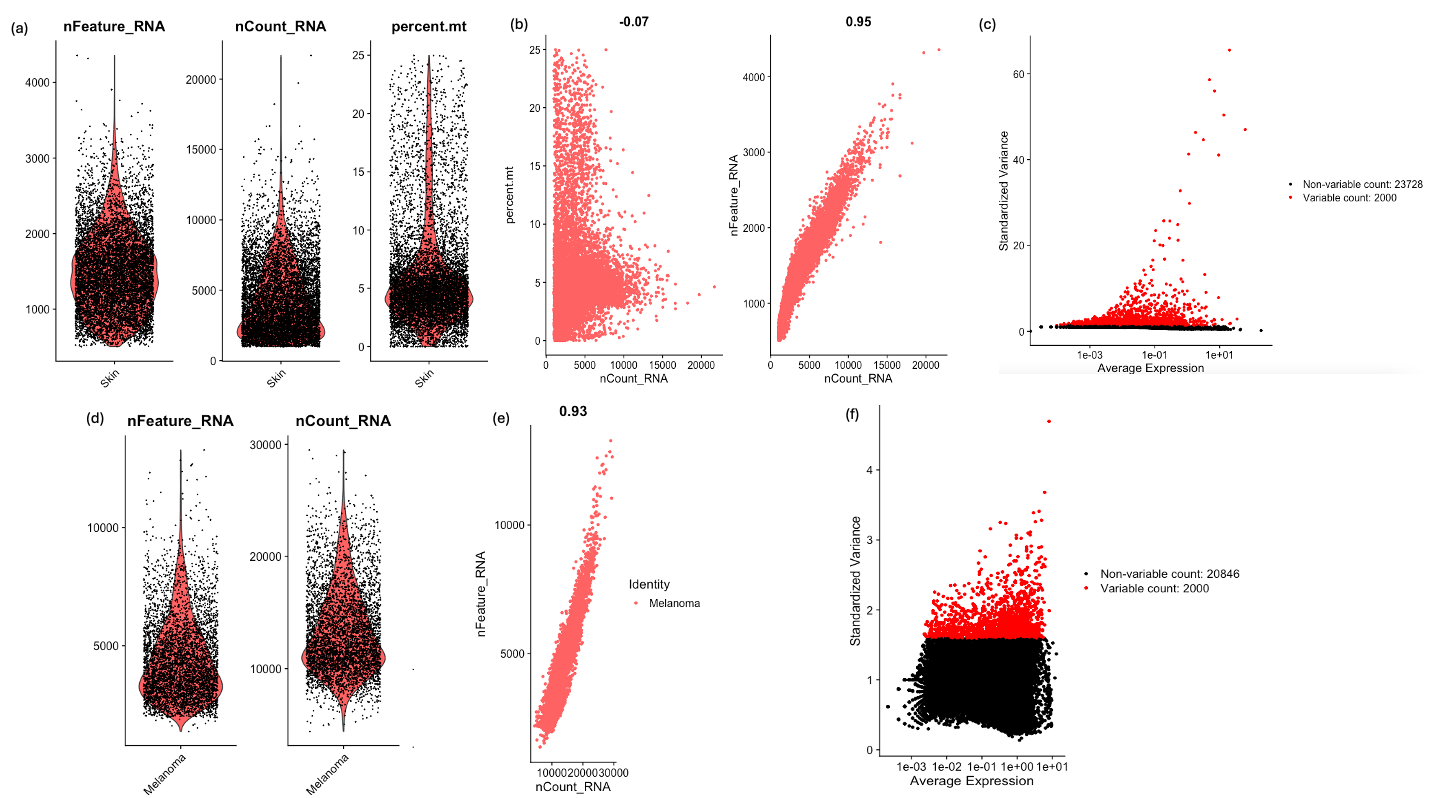


**Figure S5 QC of healthy skin and SKCM samples before the downstream analyses.** (a) Plots of QC metrics which include the number of genes, number of UMIs, and the percentage of transcripts mapping to the mitochondrial genome. Violin plots of scRNA-seq data of healthy skin sample (GSM4850587). After QC, 84,363 high-quality cells were maintained altogether for the down-stream analysis, which was illustrated as 'nFeature_RNA' and 'nCount_RNA'. (b) Sample gene plots detecting cells with deviant levels of mitochondrial transcripts and UMIs post QC. (c) Subset of features exhibiting high cell-to-cell variation in the GSM4850587 dataset was calculated by directly modelling the mean-variance relationship inherent in single-cell data, returning 2,000 features per dataset by default for PCA. (d) Violin plots of scRNA-seq data of SKCM sample. After QC, 22,846 genes across 3,700 cells were obtained after QC from 4,646 cells from the selected samples. (e) Sample gene plots detecting cells with deviant levels of UMIs post QC. (f) Mean-variance relationship inherent in single-cell data to show the subset of features exhibiting high cell-to-cell variation was calculated.

**Figure S6.**

**
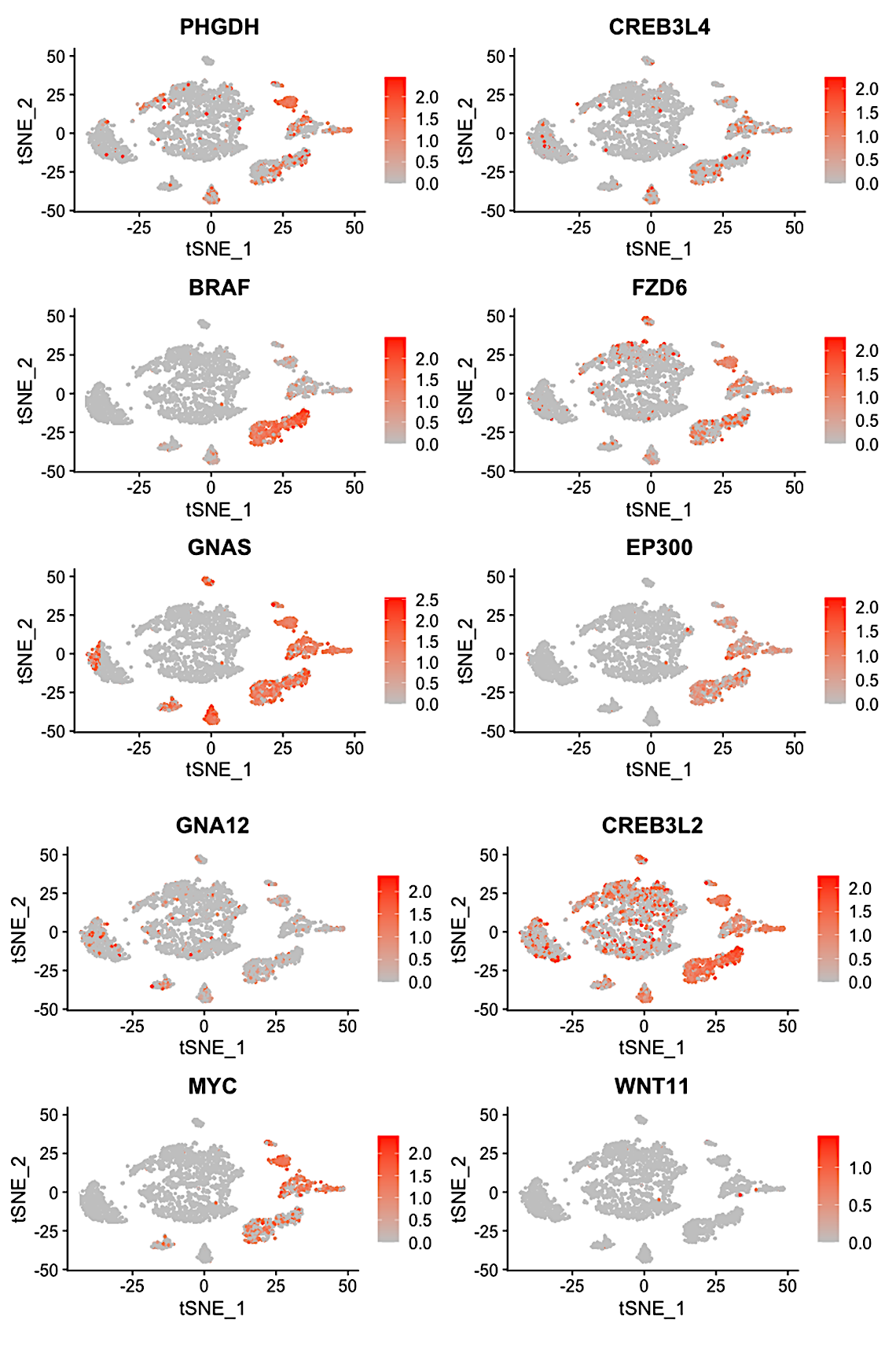
**

**Figure S6. Signature markers for tumour programmed genes in SKCM marked in the tSNE plot plotted for the SKCM analysis.** This shows which cluster group correlate with the genotypic features of TP. The markers used were based on the malignant gene expressions for SKCM according to Guan et al. (25).

**Supplementary Tables**

**Table S1 Expression levels of NRP1 and NRP2 across 19 TCGA cancer type datasets compared to ccRCC and SKCM**

| **Dunnett's multiple comparisons test** | **Mean Diff.** | **95.00% CI of diff.** | **Below threshold?** | **Adjusted P Value** | | |  |  |
| --- | --- | --- | --- | --- | --- | --- | --- | --- |
| Renal Clear Cell Carcinoma vs. Adrenocortical Carcinoma | 8042 | 7360 to 8723 | Yes | <0.0001 | | |  |  |
| Renal Clear Cell Carcinoma vs. Bladder Cancer | 8990 | 8618 to 9363 | Yes | <0.0001 | | |  |  |
| Renal Clear Cell Carcinoma vs. Breast Cancer | 8245 | 7944 to 8546 | Yes | <0.0001 | | |  |  |
| Renal Clear Cell Carcinoma vs. Cervical Cancer | 9421 | 9010 to 9831 | Yes | <0.0001 | | |  |  |
| Renal Clear Cell Carcinoma vs. Cholangiocarcinoma | 6891 | 5924 to 7858 | Yes | <0.0001 | | |  |  |
| Renal Clear Cell Carcinoma vs. Colorectal Cancer | 9537 | 9199 to 9876 | Yes | <0.0001 | | |  |  |
| Renal Clear Cell Carcinoma vs. Endometrial Cancer | 8675 | 8335 to 9015 | Yes | <0.0001 | | |  |  |
| Renal Clear Cell Carcinoma vs. Esophagogastric Cancer | 8424 | 8085 to 8762 | Yes | <0.0001 | | |  |  |
| Renal Clear Cell Carcinoma vs. Glioblastoma | 8453 | 7945 to 8961 | Yes | <0.0001 | | |  |  |
| Renal Clear Cell Carcinoma vs. Head and Neck Cancer | 8844 | 8493 to 9194 | Yes | <0.0001 | | |  |  |
| Renal Clear Cell Carcinoma vs. Leukemia | 10156 | 9662 to 10649 | Yes | <0.0001 | | |  |  |
| Renal Clear Cell Carcinoma vs. Melanoma | 9229 | 8865 to 9593 | Yes | <0.0001 | | |  |  |
| Renal Clear Cell Carcinoma vs. Non-Small Cell Lung Cancer | 7602 | 7296 to 7907 | Yes | <0.0001 | | |  |  |
| Renal Clear Cell Carcinoma vs. Pancreatic Cancer | 7380 | 6891 to 7869 | Yes | <0.0001 | | |  |  |
| Renal Clear Cell Carcinoma vs. Prostate Cancer | 8268 | 7914 to 8622 | Yes | <0.0001 | | |  |  |
| Renal Clear Cell Carcinoma vs. Renal Non-Clear Cell Carcinoma | 6794 | 6404 to 7184 | Yes | <0.0001 | | |  |  |
| Renal Clear Cell Carcinoma vs. Sarcoma | 5282 | 4851 to 5713 | Yes | <0.0001 | | |  |  |
| Renal Clear Cell Carcinoma vs. Thyroid Cancer | 7845 | 7492 to 8198 | Yes | <0.0001 | | |  |  |
|  |  |  |  |  |  |  |  |  |
| **Test details** | **Mean 1** | **Mean 2** | **Mean Diff.** | **n1** | **n2** | **q** | | **DF** |
| Renal Clear Cell Carcinoma vs. Adrenocortical Carcinoma | 10391 | 2350 | 8042 | 510 | 78 | 34.39 | | 8211 |
| Renal Clear Cell Carcinoma vs. Bladder Cancer | 10391 | 1401 | 8990 | 510 | 407 | 70.33 | | 8211 |
| Renal Clear Cell Carcinoma vs. Breast Cancer | 10391 | 2146 | 8245 | 510 | 1082 | 79.82 | | 8211 |
| Renal Clear Cell Carcinoma vs. Cervical Cancer | 10391 | 970.2 | 9421 | 510 | 294 | 66.90 | | 8211 |
| Renal Clear Cell Carcinoma vs. Cholangiocarcinoma | 10391 | 3500 | 6891 | 510 | 36 | 20.78 | | 8211 |
| Renal Clear Cell Carcinoma vs. Colorectal Cancer | 10391 | 854.0 | 9537 | 510 | 592 | 82.08 | | 8211 |
| Renal Clear Cell Carcinoma vs. Endometrial Cancer | 10391 | 1716 | 8675 | 510 | 584 | 74.43 | | 8211 |
| Renal Clear Cell Carcinoma vs. Esophagogastric Cancer | 10391 | 1967 | 8424 | 510 | 593 | 72.53 | | 8211 |
| Renal Clear Cell Carcinoma vs. Glioblastoma | 10391 | 1938 | 8453 | 510 | 160 | 48.51 | | 8211 |
| Renal Clear Cell Carcinoma vs. Head and Neck Cancer | 10391 | 1548 | 8844 | 510 | 515 | 73.61 | | 8211 |
| Renal Clear Cell Carcinoma vs. Leukemia | 10391 | 235.5 | 10156 | 510 | 173 | 60.02 | | 8211 |
| Renal Clear Cell Carcinoma vs. Melanoma | 10391 | 1162 | 9229 | 510 | 443 | 73.89 | | 8211 |
| Renal Clear Cell Carcinoma vs. Non-Small Cell Lung Cancer | 10391 | 2790 | 7602 | 510 | 994 | 72.57 | | 8211 |
| Renal Clear Cell Carcinoma vs. Pancreatic Cancer | 10391 | 3011 | 7380 | 510 | 177 | 43.99 | | 8211 |
| Renal Clear Cell Carcinoma vs. Prostate Cancer | 10391 | 2123 | 8268 | 510 | 493 | 68.07 | | 8211 |
| Renal Clear Cell Carcinoma vs. Renal Non-Clear Cell Carcinoma | 10391 | 3597 | 6794 | 510 | 348 | 50.81 | | 8211 |
| Renal Clear Cell Carcinoma vs. Sarcoma | 10391 | 5109 | 5282 | 510 | 253 | 35.71 | | 8211 |
| Renal Clear Cell Carcinoma vs. Thyroid Cancer | 10391 | 2546 | 7845 | 510 | 498 | 64.75 | | 8211 |

| **Dunnett's multiple comparisons test** | **Mean Diff.** | **95.00% CI of diff.** | **Below threshold?** | **Adjusted P Value** | | |  |  |
| --- | --- | --- | --- | --- | --- | --- | --- | --- |
| Melanoma vs. Adrenocortical Carcinoma | 5319 | 4755 to 5883 | Yes | <0.0001 | | |  |  |
| Melanoma vs. Bladder Cancer | 5743 | 5428 to 6058 | Yes | <0.0001 | | |  |  |
| Melanoma vs. Breast Cancer | 5413 | 5154 to 5671 | Yes | <0.0001 | | |  |  |
| Melanoma vs. Cervical Cancer | 5623 | 5278 to 5968 | Yes | <0.0001 | | |  |  |
| Melanoma vs. Cholangiocarcinoma | 5852 | 5057 to 6647 | Yes | <0.0001 | | |  |  |
| Melanoma vs. Colorectal Cancer | 5864 | 5576 to 6152 | Yes | <0.0001 | | |  |  |
| Melanoma vs. Endometrial Cancer | 5339 | 5049 to 5628 | Yes | <0.0001 | | |  |  |
| Melanoma vs. Esophagogastric Cancer | 5260 | 4972 to 5549 | Yes | <0.0001 | | |  |  |
| Melanoma vs. Glioblastoma | 4399 | 3976 to 4823 | Yes | <0.0001 | | |  |  |
| Melanoma vs. Head and Neck Cancer | 4636 | 4338 to 4933 | Yes | <0.0001 | | |  |  |
| Melanoma vs. Leukemia | 6428 | 6017 to 6839 | Yes | <0.0001 | | |  |  |
| Melanoma vs. Non-Small Cell Lung Cancer | 4902 | 4640 to 5164 | Yes | <0.0001 | | |  |  |
| Melanoma vs. Pancreatic Cancer | 4561 | 4153 to 4969 | Yes | <0.0001 | | |  |  |
| Melanoma vs. Prostate Cancer | 6052 | 5752 to 6353 | Yes | <0.0001 | | |  |  |
| Melanoma vs. Renal Clear Cell Carcinoma | 4858 | 4560 to 5156 | Yes | <0.0001 | | |  |  |
| Melanoma vs. Renal Non-Clear Cell Carcinoma | 6014 | 5685 to 6343 | Yes | <0.0001 | | |  |  |
| Melanoma vs. Sarcoma | 3437 | 3075 to 3798 | Yes | <0.0001 | | |  |  |
| Melanoma vs. Thyroid Cancer | 3254 | 2955 to 3554 | Yes | <0.0001 | | |  |  |
|  |  |  |  |  |  |  |  |  |
| **Test details** | **Mean 1** | **Mean 2** | **Mean Diff.** | **n1** | **n2** | **q** | | **DF** |
| Melanoma vs. Adrenocortical Carcinoma | 6475 | 1156 | 5319 | 443 | 78 | 27.39 | | 8211 |
| Melanoma vs. Bladder Cancer | 6475 | 732.1 | 5743 | 443 | 407 | 52.88 | | 8211 |
| Melanoma vs. Breast Cancer | 6475 | 1063 | 5413 | 443 | 1082 | 60.67 | | 8211 |
| Melanoma vs. Cervical Cancer | 6475 | 851.9 | 5623 | 443 | 294 | 47.26 | | 8211 |
| Melanoma vs. Cholangiocarcinoma | 6475 | 623.2 | 5852 | 443 | 36 | 21.35 | | 8211 |
| Melanoma vs. Colorectal Cancer | 6475 | 611.1 | 5864 | 443 | 592 | 59.01 | | 8211 |
| Melanoma vs. Endometrial Cancer | 6475 | 1137 | 5339 | 443 | 584 | 53.57 | | 8211 |
| Melanoma vs. Esophagogastric Cancer | 6475 | 1215 | 5260 | 443 | 593 | 52.96 | | 8211 |
| Melanoma vs. Glioblastoma | 6475 | 2076 | 4399 | 443 | 160 | 30.16 | | 8211 |
| Melanoma vs. Head and Neck Cancer | 6475 | 1839 | 4636 | 443 | 515 | 45.23 | | 8211 |
| Melanoma vs. Leukemia | 6475 | 47.15 | 6428 | 443 | 173 | 45.33 | | 8211 |
| Melanoma vs. Non-Small Cell Lung Cancer | 6475 | 1573 | 4902 | 443 | 994 | 54.25 | | 8211 |
| Melanoma vs. Pancreatic Cancer | 6475 | 1914 | 4561 | 443 | 177 | 32.43 | | 8211 |
| Melanoma vs. Prostate Cancer | 6475 | 422.7 | 6052 | 443 | 493 | 58.45 | | 8211 |
| Melanoma vs. Renal Clear Cell Carcinoma | 6475 | 1617 | 4858 | 443 | 510 | 47.29 | | 8211 |
| Melanoma vs. Renal Non-Clear Cell Carcinoma | 6475 | 461.1 | 6014 | 443 | 348 | 53.08 | | 8211 |
| Melanoma vs. Sarcoma | 6475 | 3039 | 3437 | 443 | 253 | 27.57 | | 8211 |
| Melanoma vs. Thyroid Cancer | 6475 | 3221 | 3254 | 443 | 498 | 31.50 | | 8211 |

**Table S2 Canonical gene markers for different cell types of the kidney tissue**

| **Cluster** | **Marker** |
| --- | --- |
| **Glomerular parietal epithelial cells** | *CD24, KRT8, KRT18, VCAM* |
| **Endothelial cells** | *SPARCL1, IGFBP7, CD31, CD34, ACKR1, A2M, FCN3, SPRY1, ID1, IF127, HES1* |
| **Proximal tubule** | *SLC13A3, SLC34A1, GPX3, DCXR* |
| **Proximal convoluted tubule** | *SLC22A8, PDK4, NEAT1, ACSM2A, SLC17A3, ACSM2B, SLC5A12, VMP1, PNCK* |
| **Proximal straight tubule** | *VCAM1, SLC22A7* |
| **Fibroblast** | *COL1A1, COL1A2, MGP, COL3A1, SPARC, DCN, LUM, TAGLN, BGN* |
| **CD4 CD8^+^ T cells** | *CXCL13, CCL4, TNFRSF9, CD27, NKG7, CCL3, LAG3, GZMB, GZMK, IFNG, CD8A* |
| **Macrophage** | *LYZ, CD14, CD163, HLA-D, SR, CD206, CD200R, TGM2, DECOYR, IL1R2, RETNLB, ARG1, CD86, TLR8, TLR1, CD68, AIF1* |
| **Distal tubule and collecting duct** | *DEFB1, UMOD* |
| **Collecting duct intercalated cells** | *ATP6V1G3, ATP6V0D2, TMEM213* |
| **B cells** | *CD79A, CD79B* |
| **Keratinocytes** | *KRT5, KRT14, ITGB1, ITGA6, KRT1, KRT10, SBSN, SPINK5* |
| **NK cells** | *GNLY, NKG7, CD3D, CD3E, IL7R* |
| **T-reg cells** | *LTB, TNFRSF18, IL32, TNFRSF4, BATF, S100A4, TIGIT, FOXP3, PIM2, CTLA4, PD1, LCK, CD2, CYTOR* |

**Table S3 Canonical gene markers for different cell types of the skin tissue**

| **Cluster** | **Marker** |
| --- | --- |
| **Macrophage** | *CD163, HLA-D, CD206, CD200R, TGM2, DECOYR, IL1R2, RETNLB, ARG1, CD86, TLR8, TLR1, LYZ, CD68, AIF1* |
| **Endothelial Cells** | *SELE, CLDN5, CDH5, VWF, LYVE1, PROX1* |
| **Langerhans Cells (Epithelial cells)** | *CD207, CD1A, CD11C* |
| **Keratinocyte (Epithelial cells)** | *KRT5, KRT14, TP63, ITGB1, ITGA6, KRT1, KRT10, SBSN, LOR, LG, SPINK5* |
| **Melanocytes (Epithelial cells)** | *PMEL, MLANA, TYRP1, DCT* |
| **CAF** | *ACTA2, VIM, S100A4, DES, FAP, PDGFRB, MME, CAV1* |
| **Fibroblast** | *LUM, DCN, VIM, PDGFRA, COL1A2* |
| **CD4+ CD8+ T Cells** | *CD3, CD4, CD8* |
| **T Reg Cells** | *CD3, PD1, CTLA4, CD25, CD127* |
| **B Cells** | *CD19, CD20, CD79A, CD22, CD23, CD38, CD138* |
| **NK cells** | *LYZ, CD14, GNLY, NKG7* |

**Table S4 Expression level of NRP1 across the different cluster types in both healthy kidney and ccRCC samples for comparison.** The values in each column represent the expression level of NRP1 in the corresponding cell. These values are measured in counts (transcripts per million (TPM)), indicating how actively the gene is being transcribed in each cell. Asterisks to indicate the cell types which express a higher level of *NRP1* in the ccRCC sample than the healthy kidneys sample. Asterisks indicate higher expressions of *NRP* in the tumour sample.

|  | **Healthy kidney** | **ccRCC** |
| --- | --- | --- |
| ***Glomerular parietal epithelial cells** | 0.26746372 | 0.57072408 |
| **Endothelial cells** | 0.070500737 | 1.75876965 |
| ***Distal tubule collecting duct** | 0.031464212 | 0.53397017 |
| **Proximal tubule collecting duct** | 0.031464212 | 0.01067518 |
| **Proximal straight tubules** | 0.351730677 | 0.01067518 |
| **Proximal convoluted tubule** | 0.014847529 | 0.01067518 |
| **Collecting duct intercalated cells** | 0.025059793 | 0.53397017 |
| **Tissue stem cells** | 0.040447474 | 0.00013242 |
| **T cells** | 0.049110602 | 0.00843051 |
| **Macrophage** | 0.052545121 | 0.4949393 |
| **Smooth muscle cells** | 0.869666454 | 0.14650199 |
| **Epithelial cells** | 0.076899013 | 0.57072408 |
| ***Fibroblast** | 0.042028474 | 0.32508782 |
| **NK cells** | 0.049110602 | 1.4456611 |
|  |  |  |
|  |  |  |
| **Difference** **comparison** | | |
| **Mean of differences** | 0.3178 | |
| **SD of differences** | 0.6296 | |
| **SEM of differences** | 0.1683 | |
| **95% confidence interval** | -0.04577 to 0.6813 | |

**Table S5 Expression level of NRP2 across the different cluster types in both healthy skin and SKCM samples for comparison.** The values in each column represent the expression level of *NRP2* in the corresponding cell. These values are measured in counts (transcripts per million (TPM)), indicating how actively the gene is being transcribed in each cell. Asterisks to indicate the cell types which express a higher level of *NRP2* in the SKCM sample than the healthy skin sample. Asterisks indicate higher expressions of *NRP* in the tumour sample.

|  | **Healthy Skin** | **SKCM** |
| --- | --- | --- |
| ***NK cells** | 0.01018532 | 0.03555572 |
| ***CD4^+^ CD8^+^ T cells** | 0.02476096 | 0.04307942 |
| ***B cells** | 0.005404288 | 0.06391102 |
| **CAF** | N.A. | 1.71520968 |
| ***Melanocytes** | 0.125445781 | 1.939952 |
| **Fibroblast** | 0.605331576 | 0.23235208 |
| ***Keratinocytes** | 0.076244517 | 0.71947829 |
| ***Macrophage** | 0.588200445 | 1.53831202 |
| ***Endothelial cells** | 1.277569389 | 1.6774657 |
|  |  |  |
| **Difference comparison** | | |
| **Mean of differences** | 0.4421 | |
| **SD of differences** | 0.6916 | |
| **SEM of differences** | 0.2445 | |
| **95% confidence interval** | -0.1361 to 1.020 | |

**Table S6.** **Multifaceted Gene Expression Analysis revealing NRP1- related DEGs in the extracted macrophages in ccRCC.** Top 50 DEGs are outlined for each of the NRP1^+^ and NRP1^-^ subgroups with the PCA results differentiating the heterogenous groups of cells based on the presence of NRP1, with P values and average Log2FC values.

| **DEG** | **p_val** | **avg_log2FC** | **pct.1** | **pct.2** | **p_val_adj** | **Subgroup** | **Cancer Type** |
| --- | --- | --- | --- | --- | --- | --- | --- |
| **NRP1** | 0 | 12.1823375 | 1 | 0 | 0 | NRP1+ | ccRCC |
| **DAB2** | 6.6832E-87 | 2.2060917 | 0.652 | 0.218 | 1.7195E-82 | NRP1+ | ccRCC |
| **SLCO2B1** | 1.6392E-82 | 2.18329747 | 0.579 | 0.174 | 4.2172E-78 | NRP1+ | ccRCC |
| **CTSB** | 3.2969E-69 | 1.81263019 | 0.898 | 0.672 | 8.4823E-65 | NRP1+ | ccRCC |
| **F13A1** | 8.2043E-67 | 2.03385641 | 0.602 | 0.22 | 2.1108E-62 | NRP1+ | ccRCC |
| **SELENOP** | 3.3914E-66 | 2.06432244 | 0.675 | 0.284 | 8.7254E-62 | NRP1+ | ccRCC |
| **MAF** | 1.3446E-60 | 2.14327847 | 0.461 | 0.137 | 3.4595E-56 | NRP1+ | ccRCC |
| **LGMN** | 2.0552E-57 | 1.9764542 | 0.607 | 0.251 | 5.2877E-53 | NRP1+ | ccRCC |
| **CTSL** | 2.5363E-57 | 2.09624062 | 0.691 | 0.344 | 6.5255E-53 | NRP1+ | ccRCC |
| **MSR1** | 1.8263E-54 | 1.77424275 | 0.649 | 0.294 | 4.6986E-50 | NRP1+ | ccRCC |
| **SPP1** | 1.9788E-53 | 2.29639777 | 0.827 | 0.568 | 5.0911E-49 | NRP1+ | ccRCC |
| **A2M** | 2.4438E-53 | 1.84233556 | 0.505 | 0.183 | 6.2875E-49 | NRP1+ | ccRCC |
| **FOLR2** | 1.0611E-52 | 2.08696533 | 0.469 | 0.163 | 2.73E-48 | NRP1+ | ccRCC |
| **ZFP36L1** | 1.6976E-51 | 1.37749188 | 0.835 | 0.519 | 4.3676E-47 | NRP1+ | ccRCC |
| **CD163** | 1.5897E-50 | 1.86164973 | 0.547 | 0.221 | 4.09E-46 | NRP1+ | ccRCC |
| **C1QC** | 4.7993E-50 | 1.66703295 | 0.788 | 0.496 | 1.2348E-45 | NRP1+ | ccRCC |
| **STAB1** | 4.9294E-50 | 1.91526741 | 0.448 | 0.15 | 1.2682E-45 | NRP1+ | ccRCC |
| **CD59** | 6.2883E-50 | 1.90768492 | 0.51 | 0.197 | 1.6178E-45 | NRP1+ | ccRCC |
| **PLTP** | 8.7236E-50 | 2.07341015 | 0.542 | 0.219 | 2.2444E-45 | NRP1+ | ccRCC |
| **RAP2B** | 1.0685E-48 | 1.68007787 | 0.542 | 0.218 | 2.749E-44 | NRP1+ | ccRCC |
| **LIPA** | 4.953E-48 | 1.70541526 | 0.657 | 0.333 | 1.2743E-43 | NRP1+ | ccRCC |
| **ABCA1** | 1.4917E-46 | 1.95199387 | 0.38 | 0.115 | 3.8379E-42 | NRP1+ | ccRCC |
| **TTYH3** | 3.5494E-46 | 2.10500773 | 0.369 | 0.111 | 9.132E-42 | NRP1+ | ccRCC |
| **CTSD** | 9.383E-45 | 1.39875657 | 0.851 | 0.602 | 2.4141E-40 | NRP1+ | ccRCC |
| **C1QA** | 1.1281E-44 | 1.54335221 | 0.861 | 0.627 | 2.9024E-40 | NRP1+ | ccRCC |
| **BMP2K** | 1.0839E-43 | 1.80863573 | 0.432 | 0.155 | 2.7887E-39 | NRP1+ | ccRCC |
| **MFSD1** | 3.7022E-43 | 1.49159419 | 0.592 | 0.28 | 9.5249E-39 | NRP1+ | ccRCC |
| **MERTK** | 5.8713E-43 | 2.519198 | 0.233 | 0.048 | 1.5106E-38 | NRP1+ | ccRCC |
| **MAFB** | 6.7494E-43 | 1.28773534 | 0.702 | 0.392 | 1.7365E-38 | NRP1+ | ccRCC |
| **ADAP2** | 1.2045E-42 | 1.62706932 | 0.552 | 0.25 | 3.0988E-38 | NRP1+ | ccRCC |
| **GPNMB** | 2.0056E-42 | 1.91504446 | 0.495 | 0.203 | 5.16E-38 | NRP1+ | ccRCC |
| **ADAM9** | 1.0608E-41 | 1.85665724 | 0.38 | 0.124 | 2.7291E-37 | NRP1+ | ccRCC |
| **DOCK4** | 2.0616E-41 | 2.09447773 | 0.298 | 0.08 | 5.3042E-37 | NRP1+ | ccRCC |
| **CD14** | 6.2227E-41 | 1.44136016 | 0.825 | 0.572 | 1.601E-36 | NRP1+ | ccRCC |
| **SPRED1** | 1.0997E-40 | 2.21634482 | 0.298 | 0.082 | 2.8293E-36 | NRP1+ | ccRCC |
| **PLXND1** | 1.775E-40 | 2.00406027 | 0.319 | 0.092 | 4.5667E-36 | NRP1+ | ccRCC |
| **RNASE1** | 1.9546E-40 | 2.81511596 | 0.366 | 0.127 | 5.0287E-36 | NRP1+ | ccRCC |
| **LHFPL2** | 1.1068E-39 | 1.78326543 | 0.353 | 0.112 | 2.8476E-35 | NRP1+ | ccRCC |
| **PLAU** | 1.3522E-39 | 2.18772467 | 0.346 | 0.109 | 3.4789E-35 | NRP1+ | ccRCC |
| **XIST** | 1.6653E-39 | 1.05135522 | 0.741 | 0.403 | 4.2845E-35 | NRP1+ | ccRCC |
| **C1QB** | 1.6982E-39 | 1.55174705 | 0.798 | 0.569 | 4.369E-35 | NRP1+ | ccRCC |
| **MARCKS** | 1.8283E-39 | 1.50585992 | 0.652 | 0.385 | 4.7038E-35 | NRP1+ | ccRCC |
| **MS4A4A** | 6.9006E-38 | 1.59753504 | 0.607 | 0.313 | 1.7754E-33 | NRP1+ | ccRCC |
| **FCGR2A** | 7.5135E-38 | 1.25716632 | 0.668 | 0.374 | 1.9331E-33 | NRP1+ | ccRCC |
| **MS4A7** | 6.0423E-37 | 1.24260364 | 0.796 | 0.543 | 1.5546E-32 | NRP1+ | ccRCC |
| **NPL** | 8.3872E-37 | 1.64159571 | 0.44 | 0.174 | 2.1579E-32 | NRP1+ | ccRCC |
| **HIF1A** | 1.2526E-36 | 1.39704175 | 0.573 | 0.283 | 3.2227E-32 | NRP1+ | ccRCC |
| **TREM2** | 1.7486E-36 | 1.40313197 | 0.524 | 0.233 | 4.4988E-32 | NRP1+ | ccRCC |
| **GPR34** | 2.4386E-36 | 1.69712741 | 0.442 | 0.182 | 6.2739E-32 | NRP1+ | ccRCC |
| **SLC40A1** | 2.8173E-36 | 2.19352807 | 0.322 | 0.102 | 7.2483E-32 | NRP1+ | ccRCC |
| **CLEC12A** | 1.1539E-11 | 2.19471486 | 0.045 | 0.18 | 2.9688E-07 | NRP1- | ccRCC |
| **FCN1** | 4.1005E-11 | 1.77843023 | 0.152 | 0.301 | 1.055E-06 | NRP1- | ccRCC |
| **PLAC8** | 3.4092E-09 | 1.48675058 | 0.071 | 0.192 | 8.7712E-05 | NRP1- | ccRCC |
| **RPS4Y1** | 6.4637E-09 | 1.7785167 | 0.037 | 0.144 | 0.0001663 | NRP1- | ccRCC |
| **LIMD2** | 1.6017E-08 | 1.20598258 | 0.157 | 0.288 | 0.00041209 | NRP1- | ccRCC |
| **LGALS2** | 8.9873E-08 | 2.37077579 | 0.034 | 0.125 | 0.00231224 | NRP1- | ccRCC |
| **NAPSB** | 1.7512E-07 | 1.71845666 | 0.086 | 0.195 | 0.00450553 | NRP1- | ccRCC |
| **JAML** | 4.1223E-07 | 1.07400732 | 0.126 | 0.238 | 0.01060577 | NRP1- | ccRCC |
| **FGR** | 6.169E-07 | 1.14871229 | 0.105 | 0.211 | 0.01587166 | NRP1- | ccRCC |
| **LSP1** | 1.4136E-06 | 0.43425497 | 0.288 | 0.428 | 0.03636948 | NRP1- | ccRCC |
| **ICAM3** | 5.2409E-06 | 1.46636489 | 0.063 | 0.147 | 0.13483785 | NRP1- | ccRCC |
| **CD52** | 5.6019E-06 | 0.744844 | 0.254 | 0.359 | 0.14412497 | NRP1- | ccRCC |
| **PRAM1** | 1.9919E-05 | 1.59224371 | 0.037 | 0.105 | 0.5124874 | NRP1- | ccRCC |
| **SPN** | 2.8676E-05 | 1.17720621 | 0.039 | 0.107 | 0.73778606 | NRP1- | ccRCC |
| **AC114760.2** | 3.6161E-05 | 1.49494651 | 0.047 | 0.116 | 0.93034724 | NRP1- | ccRCC |
| **TSC22D3** | 3.8147E-05 | 1.33457292 | 0.165 | 0.254 | 0.98145185 | NRP1- | ccRCC |
| **CD300E** | 5.4449E-05 | 0.97236947 | 0.058 | 0.128 | 1 | NRP1- | ccRCC |
| **LILRA5** | 6.4106E-05 | 1.43453825 | 0.055 | 0.123 | 1 | NRP1- | ccRCC |
| **CD55** | 9.4223E-05 | 1.15412259 | 0.131 | 0.21 | 1 | NRP1- | ccRCC |
| **RIPOR2** | 0.00010313 | 1.29836333 | 0.05 | 0.115 | 1 | NRP1- | ccRCC |
| **KLF2** | 0.00032145 | 0.80451191 | 0.052 | 0.113 | 1 | NRP1- | ccRCC |
| **ISG20** | 0.00042925 | 1.63676237 | 0.065 | 0.125 | 1 | NRP1- | ccRCC |
| **VSIR** | 0.00044224 | 0.63015612 | 0.291 | 0.369 | 1 | NRP1- | ccRCC |
| **LYST** | 0.00103559 | 1.05726934 | 0.086 | 0.145 | 1 | NRP1- | ccRCC |
| **SMIM25** | 0.00123004 | 0.87973116 | 0.181 | 0.25 | 1 | NRP1- | ccRCC |
| **CYTIP** | 0.00139827 | 0.76101738 | 0.126 | 0.192 | 1 | NRP1- | ccRCC |
| **WARS** | 0.00173899 | 0.87812905 | 0.107 | 0.168 | 1 | NRP1- | ccRCC |
| **LILRA2** | 0.00257109 | 0.92945653 | 0.058 | 0.107 | 1 | NRP1- | ccRCC |
| **LTA4H** | 0.00337081 | 0.71777459 | 0.207 | 0.268 | 1 | NRP1- | ccRCC |
| **FAM110A** | 0.00340935 | 1.01791276 | 0.058 | 0.104 | 1 | NRP1- | ccRCC |
| **IER5** | 0.00354085 | 0.48799374 | 0.086 | 0.142 | 1 | NRP1- | ccRCC |
| **KLF4** | 0.00369968 | 0.87416383 | 0.063 | 0.111 | 1 | NRP1- | ccRCC |
| **ADGRE5** | 0.00630339 | 0.83325181 | 0.073 | 0.12 | 1 | NRP1- | ccRCC |
| **AC004687.1** | 0.00864187 | 0.80253665 | 0.107 | 0.156 | 1 | NRP1- | ccRCC |
| **RGS2** | 0.01222442 | 0.4496673 | 0.335 | 0.385 | 1 | NRP1- | ccRCC |
| **CORO1A** | 0.01318746 | 0.52835822 | 0.44 | 0.473 | 1 | NRP1- | ccRCC |
| **CAMK1** | 0.01393568 | 0.55792934 | 0.073 | 0.116 | 1 | NRP1- | ccRCC |
| **STK17B** | 0.01412068 | 0.62670712 | 0.139 | 0.189 | 1 | NRP1- | ccRCC |
| **CRIP1** | 0.01596966 | 0.64341636 | 0.068 | 0.108 | 1 | NRP1- | ccRCC |
| **TKT** | 0.01613903 | 0.35878242 | 0.293 | 0.35 | 1 | NRP1- | ccRCC |
| **LTB** | 0.01697562 | 0.29856292 | 0.081 | 0.123 | 1 | NRP1- | ccRCC |
| **SNHG5** | 0.02067062 | 0.72434079 | 0.353 | 0.387 | 1 | NRP1- | ccRCC |
| **CD48** | 0.02091047 | 0.68426003 | 0.267 | 0.308 | 1 | NRP1- | ccRCC |
| **C19orf38** | 0.02279689 | 0.75254612 | 0.086 | 0.125 | 1 | NRP1- | ccRCC |
| **P2RY13** | 0.02323034 | 0.61972444 | 0.086 | 0.126 | 1 | NRP1- | ccRCC |
| **HIST2H2AA3** | 0.02587387 | 0.73578783 | 0.089 | 0.129 | 1 | NRP1- | ccRCC |
| **STXBP2** | 0.02790862 | 0.86570711 | 0.251 | 0.292 | 1 | NRP1- | ccRCC |
| **IRAK3** | 0.04043287 | 0.73079052 | 0.073 | 0.106 | 1 | NRP1- | ccRCC |
| **ATP2B1-AS1** | 0.05656114 | 0.42231871 | 0.092 | 0.124 | 1 | NRP1- | ccRCC |
| **TES** | 0.06692013 | 0.4361245 | 0.079 | 0.109 | 1 | NRP1- | ccRCC |

**Table S7** **Multifaceted Gene Expression Analysis revealing NRP2-related DEGs in the extracted macrophages in SKCM.** Top 50 DEGs are outlined for each of the NRP2^+^ and NRP2^-^ subgroups with the PCA results differentiating the heterogenous groups of cells based on the presence of NRP2, with P values and average Log2FC values.

| **DEG** | **p_val** | **avg_log2FC** | **pct.1** | **pct.2** | **p_val_adj** | **Subgroup** | **Cancer Type** |
| --- | --- | --- | --- | --- | --- | --- | --- |
| **NRP2** | 5.3226E-25 | 7.67439758 | 1 | 0 | 1.216E-20 | NRP2+ | SKCM |
| **C1QB** | 9.0517E-09 | 1.09095314 | 0.977 | 0.514 | 0.00020679 | NRP2+ | SKCM |
| **DAB2** | 7.6138E-07 | 1.10623033 | 0.977 | 0.542 | 0.01739444 | NRP2+ | SKCM |
| **LGMN** | 8.9718E-07 | 1.03022319 | 0.977 | 0.75 | 0.0204969 | NRP2+ | SKCM |
| **SLCO2B1** | 6.4515E-11 | 1.40500879 | 0.953 | 0.514 | 1.4739E-06 | NRP2+ | SKCM |
| **C1QC** | 4.1219E-09 | 1.12141673 | 0.953 | 0.5 | 9.417E-05 | NRP2+ | SKCM |
| **STAB1** | 9.6384E-09 | 1.24540938 | 0.93 | 0.556 | 0.0002202 | NRP2+ | SKCM |
| **APOE** | 6.7134E-07 | 1.24323126 | 0.884 | 0.444 | 0.01533749 | NRP2+ | SKCM |
| **PLTP** | 2.4252E-08 | 1.54494065 | 0.86 | 0.361 | 0.00055407 | NRP2+ | SKCM |
| **TSPAN4** | 2.7759E-08 | 1.57204887 | 0.86 | 0.319 | 0.00063418 | NRP2+ | SKCM |
| **A2M** | 5.3687E-08 | 1.35017889 | 0.837 | 0.403 | 0.00122653 | NRP2+ | SKCM |
| **LGALS3BP** | 2.453E-06 | 1.07970945 | 0.837 | 0.514 | 0.05604148 | NRP2+ | SKCM |
| **ATP1B1** | 3.5398E-06 | 1.23176121 | 0.837 | 0.333 | 0.08087079 | NRP2+ | SKCM |
| **FPR3** | 6.042E-06 | 1.11422312 | 0.837 | 0.5 | 0.13803557 | NRP2+ | SKCM |
| **GYPC** | 7.626E-08 | 1.66965967 | 0.814 | 0.264 | 0.00174223 | NRP2+ | SKCM |
| **IDH1** | 1.9224E-05 | 1.15028551 | 0.814 | 0.431 | 0.43919765 | NRP2+ | SKCM |
| **SLC1A3** | 6.2887E-05 | 1.02996443 | 0.814 | 0.431 | 1 | NRP2+ | SKCM |
| **APOBEC3A** | 0.00342904 | -1.1572066 | 0.814 | 0.792 | 1 | NRP2+ | SKCM |
| **ACP2** | 6.9952E-07 | 1.40588787 | 0.791 | 0.333 | 0.01598118 | NRP2+ | SKCM |
| **NRP1** | 8.6772E-05 | 1.0552464 | 0.791 | 0.361 | 1 | NRP2+ | SKCM |
| **CCDC47** | 0.00010295 | 1.03701919 | 0.791 | 0.389 | 1 | NRP2+ | SKCM |
| **VAT1** | 0.00018028 | 1.02580348 | 0.791 | 0.403 | 1 | NRP2+ | SKCM |
| **METTL16** | 0.00029024 | 1.07171444 | 0.791 | 0.542 | 1 | NRP2+ | SKCM |
| **SIRT5** | 0.00058752 | 1.036442 | 0.791 | 0.472 | 1 | NRP2+ | SKCM |
| **SLC27A1** | 0.00125399 | 1.06558834 | 0.791 | 0.569 | 1 | NRP2+ | SKCM |
| **ETV5** | 3.0576E-05 | 1.18844509 | 0.767 | 0.333 | 0.69853704 | NRP2+ | SKCM |
| **ADAM28** | 5.2547E-05 | 1.18928607 | 0.767 | 0.417 | 1 | NRP2+ | SKCM |
| **C21orf33** | 6.6221E-05 | 1.09473469 | 0.767 | 0.417 | 1 | NRP2+ | SKCM |
| **PRPF8** | 0.00013236 | 1.03685662 | 0.767 | 0.389 | 1 | NRP2+ | SKCM |
| **CD59** | 0.00013803 | 1.09980406 | 0.767 | 0.403 | 1 | NRP2+ | SKCM |
| **SLC25A45** | 0.00014533 | 1.26105946 | 0.767 | 0.514 | 1 | NRP2+ | SKCM |
| **MAF** | 0.00016028 | 1.01430784 | 0.767 | 0.347 | 1 | NRP2+ | SKCM |
| **FUCA1** | 1.1527E-06 | 1.56882644 | 0.744 | 0.278 | 0.02633427 | NRP2+ | SKCM |
| **SDC3** | 1.3721E-06 | 1.54751772 | 0.744 | 0.25 | 0.03134622 | NRP2+ | SKCM |
| **SIGLEC1** | 8.0497E-05 | 1.09360272 | 0.744 | 0.444 | 1 | NRP2+ | SKCM |
| **BTN3A1** | 0.00025229 | 1.16942415 | 0.744 | 0.444 | 1 | NRP2+ | SKCM |
| **CD72** | 0.0003985 | 1.05593817 | 0.744 | 0.389 | 1 | NRP2+ | SKCM |
| **IGF1** | 0.00119326 | 1.13193084 | 0.744 | 0.556 | 1 | NRP2+ | SKCM |
| **AMBRA1** | 0.00234194 | 1.02745321 | 0.744 | 0.542 | 1 | NRP2+ | SKCM |
| **PMP22** | 1.2364E-09 | 2.31856908 | 0.721 | 0.139 | 2.8247E-05 | NRP2+ | SKCM |
| **PLBD2** | 3.3781E-07 | 1.78630458 | 0.721 | 0.292 | 0.0077175 | NRP2+ | SKCM |
| **GPR34** | 5.3969E-06 | 1.43699891 | 0.721 | 0.278 | 0.12329813 | NRP2+ | SKCM |
| **GALC** | 5.9775E-06 | 1.49168103 | 0.721 | 0.375 | 0.1365629 | NRP2+ | SKCM |
| **CEPT1** | 7.0852E-06 | 1.47517141 | 0.721 | 0.292 | 0.16186802 | NRP2+ | SKCM |
| **AP2B1** | 1.7294E-05 | 1.24770275 | 0.721 | 0.264 | 0.39509986 | NRP2+ | SKCM |
| **NAA20** | 3.1524E-05 | 1.27203922 | 0.721 | 0.333 | 0.72019473 | NRP2+ | SKCM |
| **C1orf85** | 0.00011973 | 1.12448203 | 0.721 | 0.319 | 1 | NRP2+ | SKCM |
| **TCF4** | 0.0002103 | 1.06051735 | 0.721 | 0.389 | 1 | NRP2+ | SKCM |
| **POM121** | 0.00043466 | 1.1289504 | 0.721 | 0.431 | 1 | NRP2+ | SKCM |
| **SLC47A1** | 0.00124028 | 1.05853358 | 0.721 | 0.458 | 1 | NRP2+ | SKCM |
| **SLC17A5** | 0.00324704 | 1.01076432 | 0.721 | 0.542 | 1 | NRP2+ | SKCM |
| **CRIP1** | 4.7903E-05 | 2.64004405 | 0.07 | 0.431 | 1 | NRP2- | SKCM |
| **FGR** | 5.3723E-05 | 1.10242707 | 0.512 | 0.722 | 1 | NRP2- | SKCM |
| **CD52** | 0.00025424 | 1.75660509 | 0.186 | 0.486 | 1 | NRP2- | SKCM |
| **FPR2** | 0.00028716 | 1.96683314 | 0.116 | 0.444 | 1 | NRP2- | SKCM |
| **VCAN** | 0.00061481 | 1.24446565 | 0.372 | 0.653 | 1 | NRP2- | SKCM |
| **AQP9** | 0.00104341 | 1.65269269 | 0.233 | 0.5 | 1 | NRP2- | SKCM |
| **S100A8** | 0.00112412 | 1.33378384 | 0.279 | 0.528 | 1 | NRP2- | SKCM |
| **ANPEP** | 0.00260065 | 1.38804543 | 0.209 | 0.486 | 1 | NRP2- | SKCM |
| **RAB24** | 0.00322898 | 1.23985163 | 0.256 | 0.5 | 1 | NRP2- | SKCM |
| **C19orf59** | 0.00330907 | 2.07576751 | 0.07 | 0.306 | 1 | NRP2- | SKCM |
| **S100A12** | 0.00353377 | 3.41284424 | 0.023 | 0.222 | 1 | NRP2- | SKCM |
| **CFP** | 0.00456309 | 1.86878299 | 0.14 | 0.361 | 1 | NRP2- | SKCM |
| **PRAM1** | 0.00809278 | 1.16047974 | 0.279 | 0.5 | 1 | NRP2- | SKCM |
| **CLEC4D** | 0.00846382 | 1.77091293 | 0.093 | 0.306 | 1 | NRP2- | SKCM |
| **SH2D3C** | 0.0084788 | 2.34380259 | 0.07 | 0.264 | 1 | NRP2- | SKCM |
| **APOBEC3B** | 0.00923999 | 2.54174197 | 0.023 | 0.194 | 1 | NRP2- | SKCM |
| **ZC3H12A** | 0.01077996 | 1.70803572 | 0.116 | 0.319 | 1 | NRP2- | SKCM |
| **FLNA** | 0.0108095 | 1.04293612 | 0.395 | 0.583 | 1 | NRP2- | SKCM |
| **TACC3** | 0.01098168 | 1.76730167 | 0.047 | 0.236 | 1 | NRP2- | SKCM |
| **ADAM19** | 0.01107957 | 2.53644767 | 0.07 | 0.25 | 1 | NRP2- | SKCM |
| **CBFA2T3** | 0.01117105 | 4.01122726 | 0 | 0.139 | 1 | NRP2- | SKCM |
| **FAM65B** | 0.01241474 | 1.35255507 | 0.558 | 0.667 | 1 | NRP2- | SKCM |
| **MGST1** | 0.01263504 | 2.865149 | 0.023 | 0.181 | 1 | NRP2- | SKCM |
| **CD300E** | 0.01587633 | 1.24184018 | 0.628 | 0.681 | 1 | NRP2- | SKCM |
| **APOBEC3A-B** | 0.01659123 | 4.71577137 | 0 | 0.125 | 1 | NRP2- | SKCM |
| **LIPN** | 0.01723084 | 3.61389176 | 0.023 | 0.167 | 1 | NRP2- | SKCM |
| **RHOBTB2** | 0.01723739 | 3.38562277 | 0.023 | 0.167 | 1 | NRP2- | SKCM |
| **ZBTB16** | 0.01829298 | 1.73027094 | 0.093 | 0.264 | 1 | NRP2- | SKCM |
| **LGALS2** | 0.01853417 | 1.86931663 | 0.093 | 0.264 | 1 | NRP2- | SKCM |
| **IL1RN** | 0.02011509 | 1.22100668 | 0.372 | 0.486 | 1 | NRP2- | SKCM |
| **ATP1A2** | 0.0207254 | 1.0218745 | 0.209 | 0.417 | 1 | NRP2- | SKCM |
| **HIST2H2BE** | 0.02338257 | 1.68518305 | 0.07 | 0.236 | 1 | NRP2- | SKCM |
| **CD3D** | 0.02458932 | 3.95677947 | 0 | 0.111 | 1 | NRP2- | SKCM |
| **GLT1D1** | 0.02458932 | 3.77990171 | 0 | 0.111 | 1 | NRP2- | SKCM |
| **EDARADD** | 0.02459514 | 2.42626475 | 0 | 0.111 | 1 | NRP2- | SKCM |
| **FAM172A** | 0.02610937 | 1.30364547 | 0.163 | 0.347 | 1 | NRP2- | SKCM |
| **ICAM3** | 0.0269824 | 1.71577137 | 0.116 | 0.278 | 1 | NRP2- | SKCM |
| **BCL11A** | 0.02797227 | 1.36067641 | 0.07 | 0.236 | 1 | NRP2- | SKCM |
| **RPH3A** | 0.02801644 | 2.64865718 | 0.023 | 0.153 | 1 | NRP2- | SKCM |
| **TSPAN32** | 0.03013769 | 2.34380259 | 0.023 | 0.153 | 1 | NRP2- | SKCM |
| **CDA** | 0.03051165 | 1.73207318 | 0.07 | 0.222 | 1 | NRP2- | SKCM |
| **VSTM1** | 0.03055324 | 2.2259661 | 0.023 | 0.153 | 1 | NRP2- | SKCM |
| **LINC00116** | 0.03181965 | 1.05280636 | 0.186 | 0.375 | 1 | NRP2- | SKCM |
| **PID1** | 0.03204568 | 1.75884009 | 0.093 | 0.25 | 1 | NRP2- | SKCM |
| **HMBS** | 0.03493699 | 1.35149699 | 0.047 | 0.194 | 1 | NRP2- | SKCM |
| **DHRS4L1** | 0.03633272 | 2.71577137 | 0 | 0.097 | 1 | NRP2- | SKCM |
| **CD3E** | 0.03639716 | 3.50426727 | 0 | 0.097 | 1 | NRP2- | SKCM |
| **ZBTB18** | 0.03639931 | 2.84130225 | 0 | 0.097 | 1 | NRP2- | SKCM |
