# Supplementary material for: Differential neuropilin isoform expressions highlight plasticity in macrophages in the heterogenous TME through in-silico profiling
Source: Front Immunol. 2025 Mar 11;16:1547330. doi: 10.3389/fimmu.2025.1547330 (PMC11933088; doi:10.3389/fimmu.2025.1547330)
Supplement: Supplementary file 2 [file DataSheet2.docx]

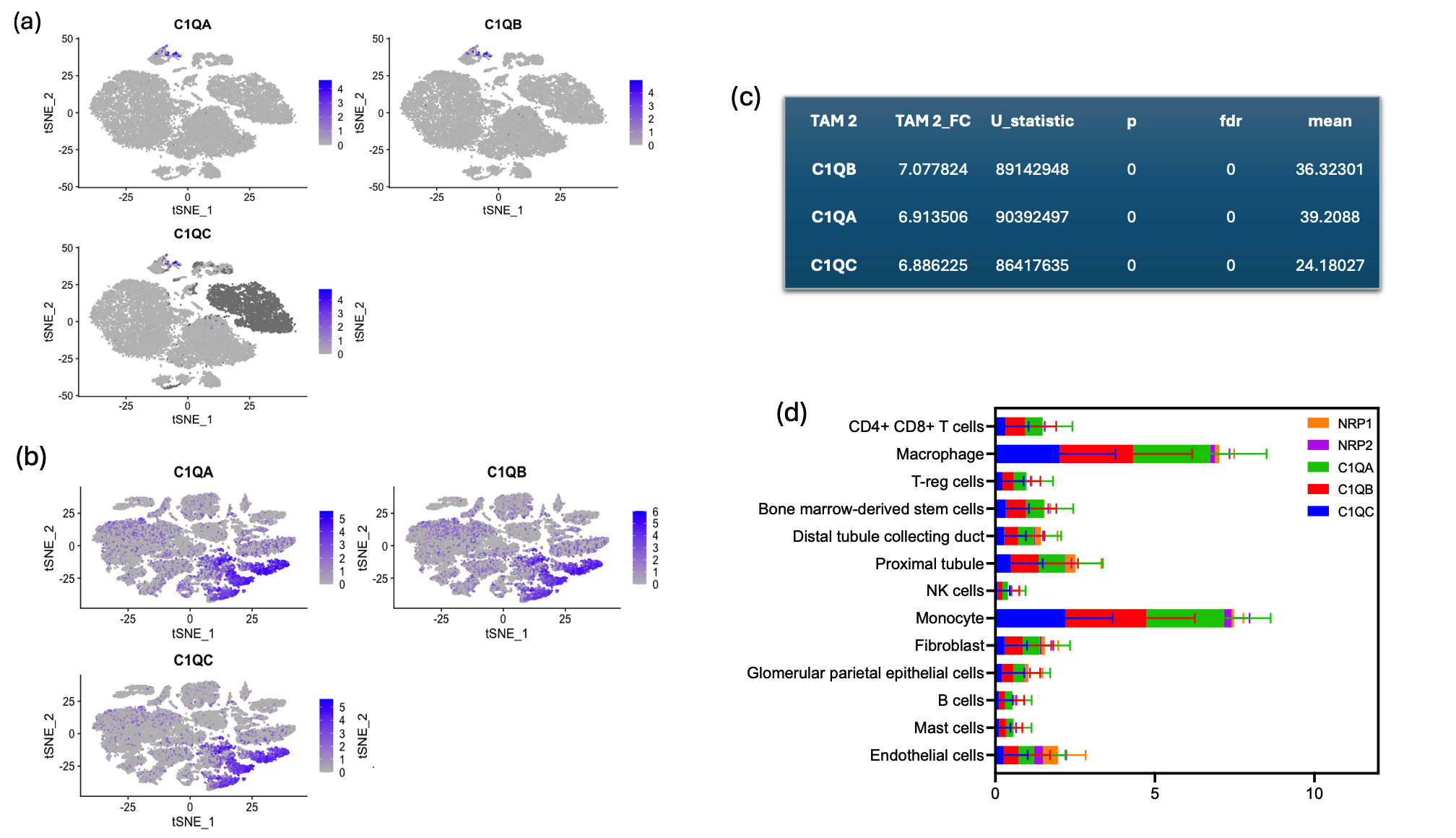


**Figure S7. Signature markers for the polarised Type 2-TAM, C1Q genes.** (a) *C1QA, C1QB,* and *C1QC* enrichment in the healthy kidney samples. (b) C1QA, C1QB, and C1QC enrichment in the ccRCC sample. (c) Expression levels of the *C1Q* genes in TAM2 following tSNE which were the top 5 genes highly expressed. (d) Macrophage shows a high level of the *C1Q* genes amongst the different cell types.

**Figure S8**.

(a)


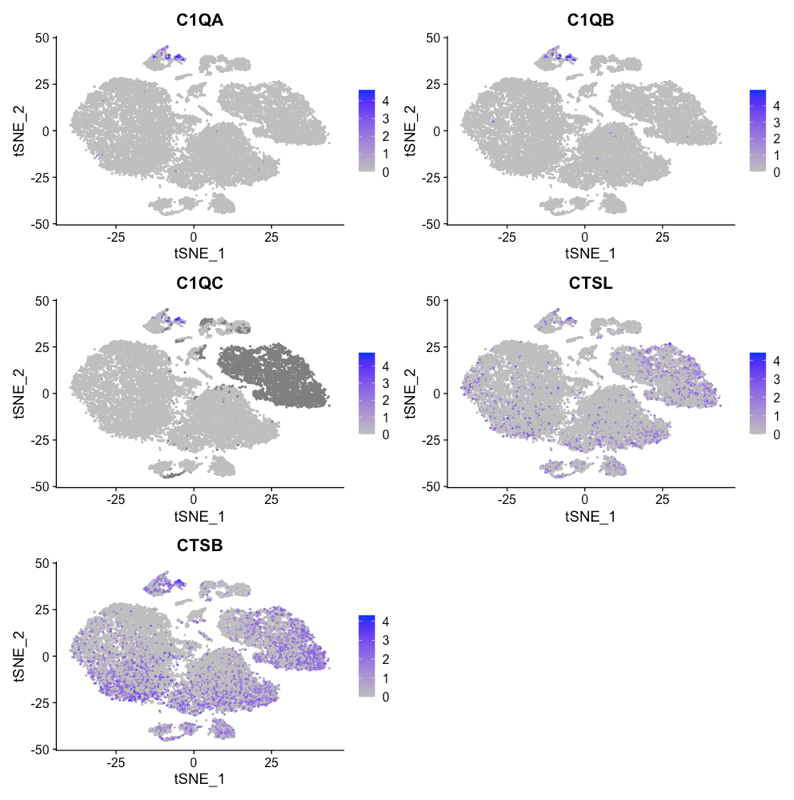

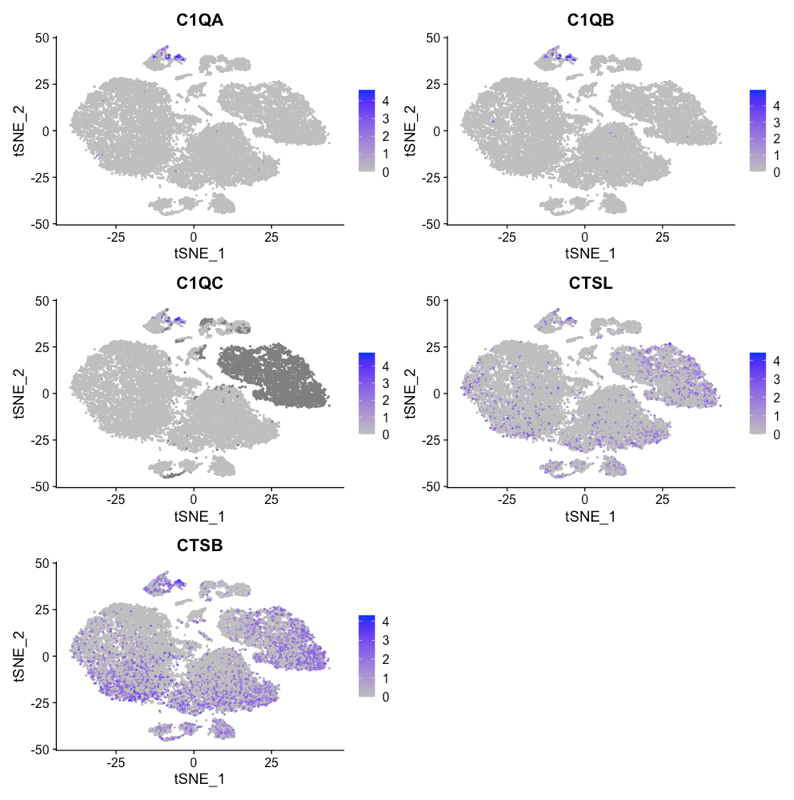


(b)


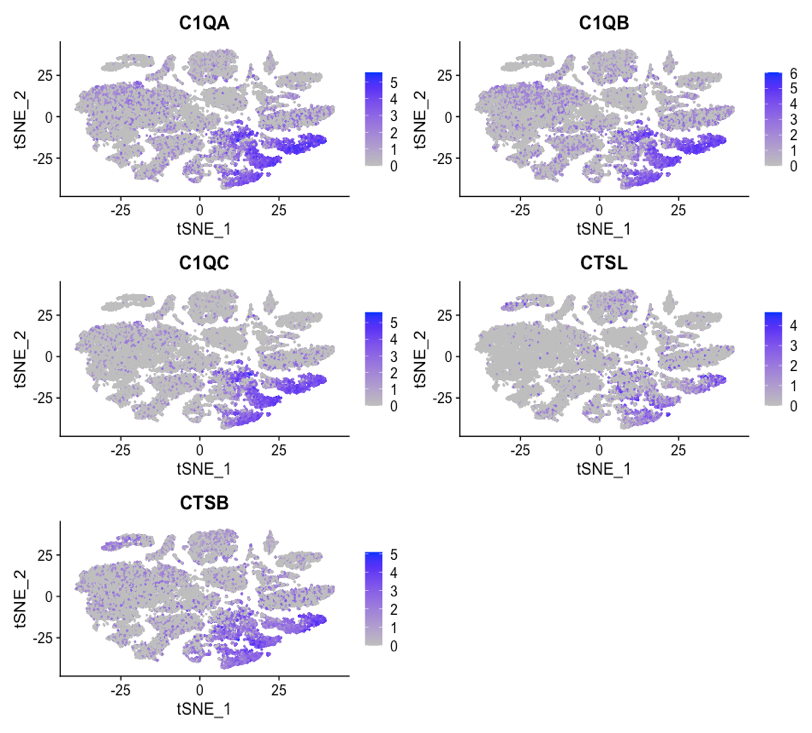

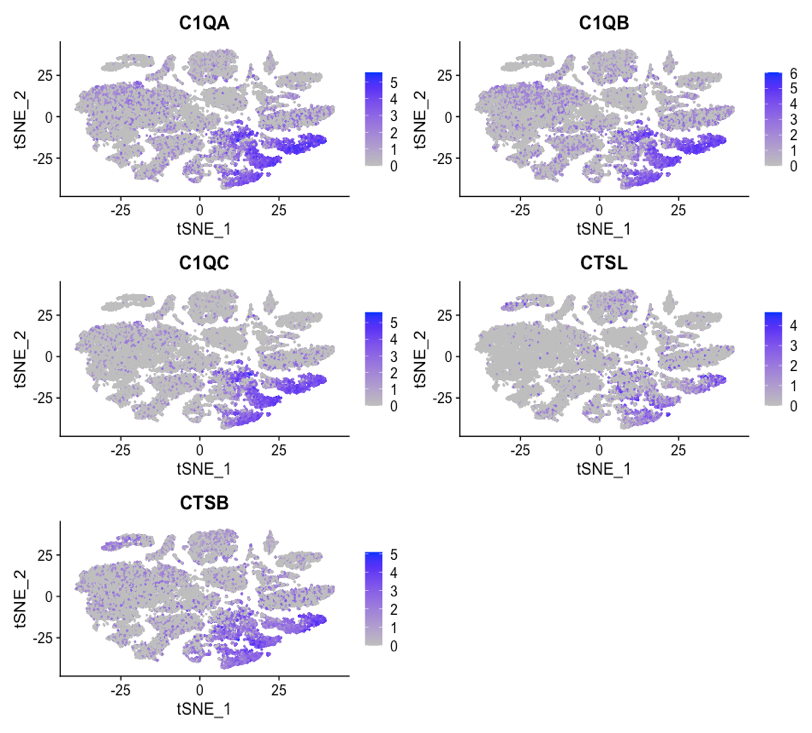


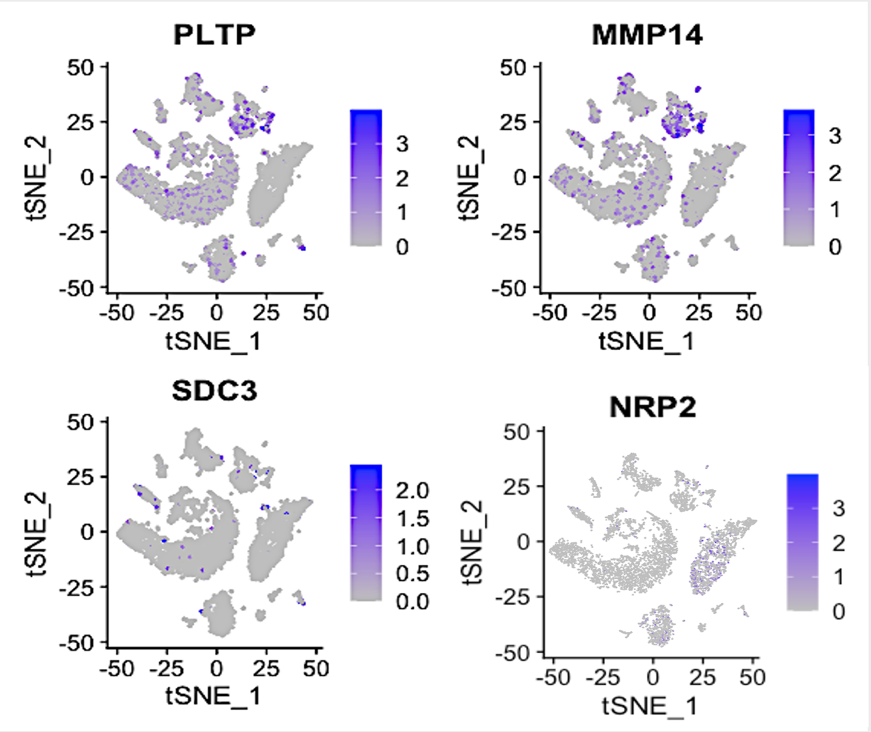
(c)


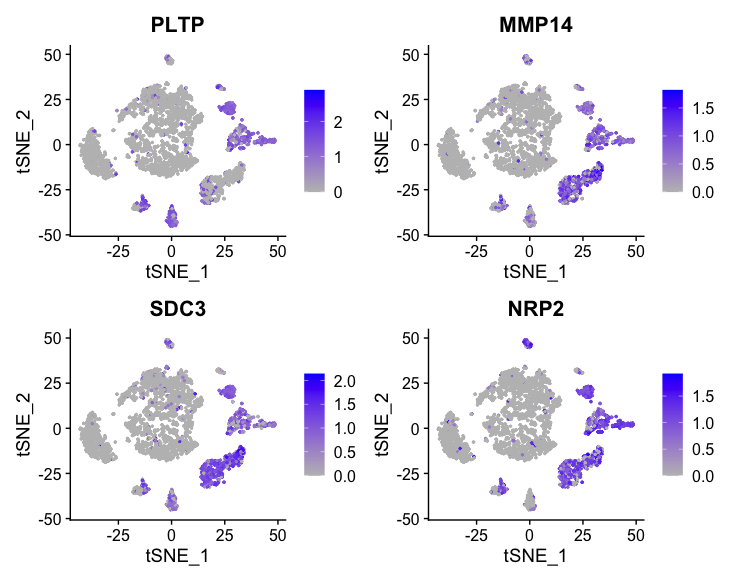
(d)

**Figure S8 Expressions of the genes associated with *NRP1* and *NRP2* in ccRCC and SKCM at the single-cell level.** **(a)** tSNE visualizations of healthy kidney (upper panel); and (**b**) clear cell renal cell carcinoma (ccRCC) samples (lower panel) illustrate the expression patterns of *CTSB* and *CTSL* across different cell types. The data suggest a higher expression of genes associated with NRP1 in the tumor microenvironment. **(c)** tSNE visualizations of healthy skin (upper panel); and **(d)** skin cutaneous melanoma (SKCM) samples (lower panel) show the expression patterns of *PLTP, MMP14*, and *SDC3* across various cell types, indicating higher expression of genes associated with *NRP2* in the tumor environment.
